# A Comprehensive Review of Phytonutrients as a Dietary Therapy for Obesity

**DOI:** 10.3390/foods12193610

**Published:** 2023-09-28

**Authors:** Shahida Anusha Siddiqui, Iskandar Azmy Harahap, Priyanka Suthar, Yuan Seng Wu, Nibedita Ghosh, Roberto Castro-Muñoz

**Affiliations:** 1 Department of Biotechnology and Sustainability, Technical University of Munich, Essigberg 3, 94315 Straubing, Germany; 2 German Institute of Food Technologies (DIL e.V.), Prof.-von-Klitzing Str. 7, 49610 Quakenbrück, Germany; 3 National Research and Innovation Agency (BRIN), Jakarta 10340, Indonesia; iskandar.azmy.harahap@brin.go.id; 4 Department of Food Science and Technology, Dr. Y. S. Parmar University of Horticulture and Forestry, Solan 173230, Himachal Pradesh, India; sutharpriyanka709@gmail.com; 5 School of Medical and Life Sciences, Sunway University, Subang Jaya 47500, Malaysia; sengwu_21@yahoo.com; 6 Department of Pharmacology, Girijananda Chowdhury University, Guwahati 781017, Assam, India; nibeditaghosh998@gmail.com; 7 Tecnologico de Monterrey, Campus Toluca, Av. Eduardo Monroy Cárdenas 2000, San Antonio Buenavista, Toluca de Lerdo 50110, Mexico; 8 Department of Sanitary Engineering, Faculty of Civil and Environmental Engineering, Gdansk University of Technology, G. Narutowicza St. 11/12, 80-233 Gdansk, Poland

**Keywords:** phytonutrients, anti-obesity, natural products, adipogenesis, dietary supplements

## Abstract

Obesity is a complex medical condition mainly caused by eating habits, genetics, lifestyle, and medicine. The present study deals with traditional diets like the Mediterranean diet, Nordic diet, African Heritage diet, Asian diet, and DASH, as these are considered to be sustainable diets for curing obesity. However, the bioavailability of phytonutrients consumed in the diet may vary, depending on several factors such as digestion and absorption of phytonutrients, interaction with other substances, cooking processes, and individual differences. Hence, several phytochemicals, like polyphenols, alkaloids, saponins, terpenoids, etc., have been investigated to assess their efficiencies and safety in the prevention and treatment of obesity. These phytochemicals have anti-obesity effects, mediated via modulation of many pathways, such as decreased lipogenesis, lipid absorption, accelerated lipolysis, energy intake, expenditure, and preadipocyte differentiation and proliferation. Owing to these anti-obesity effects, new food formulations incorporating these phytonutrients were introduced that can be beneficial in reducing the prevalence of obesity and promoting public health.

## 1. Introduction

Obesity is a condition where the body experiences metabolic changes that may cause numerous types of stomach and digestive issues characterized by altered gut flora, inflammation, and intestinal barrier disruption [1]. Obesity can also develop due to numerous interactions involving inheritable, social, and environmental factors. The prevalence of obesity is affected by dietary selection, urban development, and lifestyle [2]. Obesity has a more detrimental effect on longevity in young children and adolescents than adults.

To define an individual of both sexes (male and female) at range of its age to be obese is usually based on body mass index (BMI), where WHO considers an ideal BMI to be 18.5–24.9 by engaging everyday, ideally vigorous, physical exercise (WHO assessed on 26 June 2023). Obesity is associated with an increased chance of developing several well-known cancers, especially liver, pancreatic, kidney, gallbladder, uterine, breast, and colorectal cancer [3]. Obesity is thought to be associated with 4–8% of cancer cases [3]. The prevalence of obesity was shown in the case of meta-analysis investigation, where nearly 37 papers were included and observed that the elevated childhood body mass index (BMI) was linked to an increased risk of adult diabetes (odds ratio OR 1.70; 95%, confidence interval CI 1.30–2.22), coronary heart disease (odds ratio OR 1.20; 9%), and several malignancies, excluding breast cancer or stroke [4]. However, compared to the overall population of physically unfit, obese people, up to 30% of patients classified as obese were found to have lower gut fat content, thinner carotid intima-media, and insulin sensitivity comparable to healthy average-weight humans. The condition known as metabolic syndrome (clustering of abdominal obesity, dyslipidemia, hyperglycemia, and hypertension) has an average prevalence of 31% and increases the risk of cardiovascular disease, cerebrovascular disease, and overall mortality by 1.5 to 2 times, respectively [5]. Relying on plant-based foods, such as fruits, vegetables, and fiber-rich cereals and breads, includes a wide range of phytonutrients, such as vitamins, carotenoids, polyphenols, curcuminoids, polyunsaturated fatty acids, proteins, peptides, dietary fibers, oligosaccharides, and minerals. As a result, phytonutrients in the diet are beneficial and necessary for human health for maintaining a healthy lifestyle [6,7].

Nutrient bioavailability varies depending on a wide range of factors, including the different matrices in which nutrients are present with their chemical nature, binding form, additional foods and their constituents that may enhance or block absorption, post-absorption metabolization, host-associated variables such as health status, inheritance, and age-related habits, as well as individual factors [8]. The bioavailability of the phytonutrient component is also affected by the dietary matrix material and its dispersion through matrix-based alterations during digestion, absorption, metabolism, and distribution throughout the body. It effectively increases the lower oral bioavailability of weakly water-soluble medicines via lipid-based drug delivery systems (LbDDSs) [8]. To understand the pharmacokinetic, in vitro lipolysis analysis and SPECT/*CT,* in vivo imaging experiments were used to examine the oral absorption of fenofibrate (FF) (natural source from beans, apricots, and apples) from LbDDSs in rodents. Utilizing SPECT/CT imaging, it was demonstrated that even 24 h after delivery, the animal’s stomach contained large levels of the drug and lipid formulation despite absorption improvement, which can be one of the approaches for providing nutrients to the body in order to maximize the health advantages of phytonutrients [9]. The cell membrane’s ability to absorb nutrients also depends on their solubility; as a result, nutrients must be in their soluble form, whereas, the case of releasing insoluble salts by the nutrients, such as chelates with phytic acid, gradually reduces their bioavailability [10,11]. For instance, phytic acid in millet can potentially bind to calcium, zinc, and iron to generate insoluble complexes [12]. Thus, it necessitates that the nutrients be in their soluble form for absorption.

The combination of dietary fat consumption, hormonal changes, and other chronic diseases is one example of the many variables that lead to obesity. Obesity does not depend on the cause led by misinformation and lack of self-control in an individual over their dietary habits. Obesity may be caused due to external or internal factors depending on several factors, including social, physical, and environmental. Environmental factors, such as the availability of food, poor diets, and physical inactivity, management factors (such as the inability to control one’s lifestyle and unhealthy routines), and time factors (such as eating and sleeping patterns) are primarily the reason for the rising rates of obesity [13,14].

Food and nutrition are the primary focuses of obesity therapy since maintaining a healthy weight can be difficult. To prevent obesity, one must continue to modify their intake of nutrients, including protein and legumes, as well as oligosaccharides, polysaccharides, and fiber. Another approach is incorporating vital nutrients into the diet instead of lowering the energy associated with a limited amount of food items [14,15]. Encouraging healthy living practices requires more physical activity that reduces calorie intake and fat content. Since chronic disorders can make obesity worse, they can be controlled by maintaining the homeostasis of our body [16]. When chronic disease prevalence rates rise, the probability of undergoing productivity decreases and growing well-being expenses increase [17].

Current research is based on secondary metabolites with exhibited anti-obesity effects, including phenolics, flavonoids, and terpenoids, along with nobiletin from citrus peel, curcumin from turmeric, and anthocyanins from *Hibiscus sabdariffa* that act as powerful antioxidants [18], which may be of great importance in controlling lipid profiles and preventing severe consequences of lipid disorders. A diet high in fruits and vegetables, low in fat, and high in fiber can enhance lipid metabolism and help to manage several disorders since plant-based nutrients provide a wide variety of therapeutic effects [19]. A significant relationship between alterations in body composition, disease prevalence, and an increase in phytonutrient intake, indicating phytonutrients, as well as carbohydrates, proteins, and lipids, can help individuals achieve these advantages. These are the prime factors in reducing the prevalence of diseases like obesity [20]. Enhancing the phytonutrient content of fruits, vegetables, and other nutrients along with our understanding of their needs, consumer preferences, and mechanisms of action can be one of the other strategies to control obesity. These findings help them compete better with processed junk food, which is a major contributor to world obesity. As estimated by the World Health Organization (WHO), 600 million individuals worldwide are considered obese individuals [21]. The enhancement of phytonutrients is the focus of several studies that aim to manage the worldwide obesity crisis that drives the growing rate of obesity [22]. The accumulation of excess or abnormal fat, known as obesity, makes it difficult to maintain a healthy weight. A surplus of macronutrients in adipose tissues causes them to produce inflammatory mediators, including tumor necrosis factor and interleukin-6, and decreases the generation of adiponectin, which increases the risk of oxidative stress and a pro-inflammatory state [23]. According to research, phytochemicals can alter pro-inflammatory genes that work by limiting their expression while activating anti-inflammatory genes. This differential gene regulation is regulated by epigenetic changes. By modifying the amounts of pro-inflammatory microRNAs (miRNAs), particularly those that are elevated after nuclear factor-B (NF-B) activation, the researchers in this study demonstrate how phytochemicals might decrease inflammation. These phytochemicals also alter the main inflammatory signaling pathways, such as nuclear factor erythroid 2-related factor 2 (Nrf2), signal transducers and activators of transcription (STAT), and mitogen-activated protein kinases (MAPKs) [24]. The discussion is based on the current scenario of phytonutrients mentioned and that they can potentially act against obesity.

## 2. Phytonutrients in Regional Diet

### 2.1. Mediterranean Diet

In the early 1960s, the Mediterranean diet (MD) was described using the ensuing general characteristics. A large variety of plant-based foods, particularly those that are organically produced and abundant in nutrients (such as fruit, vegetables, bread, other cereals, potatoes, beans, nuts, and seeds); fresh fruit as the conventional standard meal; dairy-based products (mainly cheese and yogurt) that are often consumed in small to moderate amounts; whereas, olive oil as the primary source of fat and consumption of fish and poultry in small to moderate amounts are included in this diet [25]. This diet had modest levels of saturated fats as compared to the total fat intake, around 7–8%; nevertheless, given the area analyzed, total fat consumption varied significantly from less than 20% to more than 35% of the total calorie consumption. The Mediterranean diet (MD), which emphasizes a wide variety of plant foods and high polyphenol intake, is known to lower the risk of obesity through dietary patterns [25]. The essential consumption of extra-virgin olive oil (EVOO), vegetables, legumes, fruits, and whole-grain cereals has an impact on body weight by reducing lipid metabolism, oxidative stress, platelet aggregation, and coagulation [26]. The Mediterranean diet resembles some aspects of the traditional dietary practices of the various countries bordering the Mediterranean Sea [27] (Table 1).

The Mediterranean diet can assist in maintaining a healthy diet [28]. The biological components of EVOO that give it its therapeutic properties include fatty acids related to triacylglycerols, free fatty acids, and mono- and diacylglycerols. A variety of lipids like hydrocarbons, sterols, aliphatic alcohols, and tocopherols, as well as volatile compounds like polyphenols have the ability to lower the levels of oxidative stress, overweight and obese total fat in the body, and cholesterol that form in response to insect wounds in olive trees [29]. The main source of olive polyphenols is olive leaves, which are found in various olive tree parts and have the highest oxidative and scavenger capabilities. Fresh olive juice, or EVOO, is only produced by mechanical and physical methods [30]. In contrast, the smallest portion (about 2% of the weight) comprises an extensive set of little substances containing over 230 chemical compounds (aliphatic and triterpene alcohols, sterols, hydrocarbons, volatile compounds, and antioxidants) [31]. It primarily comprises mono- and polyunsaturated fatty acids, accounting for over 98% of the overall weight. Oleuropeoides, flavones, flavonols, flavan-3-ols, and substituted phenols are among the five distinct compounds in olive leaves, including phenolic. Polyphenolic compounds are particularly abundant in EVOO and have a range of advantageous biological effects, including anti-oxidative, anti-inflammatory, antiproliferative, and finally, anti-obesity and hypoglycemic action [32].

### 2.2. Nordic Diet

The Nordic diet includes additional ingredients like fish, reduced-fat dairy products, potatoes, and vegetable fat, especially apples, pears, berries, root and cruciferous vegetables, cabbage, and whole-grain and rye bread. It is distinguished by its concentration on the intake of healthy regionally specific diets, similar to the MD [33]. The dietary strategy depends on the conventional Okinawan diet [34]. The Okinawan diet is a fiber-rich diet inspired by the nutritional habits of the inhabitants of the Ryukyu Islands, wherein the median lifespan is quite substantial and nearly more. The food is based on traditional Nordic raw food and provides moderately low carbohydrate energy content with higher contents of fiber, fat, and protein [35].

However, it was modified into meals that complemented the Nordic diet and had flavors and nutrients appropriate for Nordic citizens. Traditional Nordic diets heavily influence the food plan, with the inclusion of high-fat content, chicken, fruits, berries, and nuts at its core. Additionally, there are limitations on consuming dairy goods, sugar, white flour, red meat, and chicken that has been processed. The number of foods with a high glycemic index (GI), such as white wheat and sugar, was significantly decreased in favor of nutrients that had minimal impact on blood sugar, effectively supporting lower glycemic activity. With a daily calorie intake of around 1900 kcal, this diet provides a healthy quantity of nutrients such as carbohydrates, proteins, and fats. It stimulates a low glycemic action, the contented of high glycemic index, lower effect, and blood sugar level [36] (Table 1).

The typical daily consumption of grains (wheat, rye, barley, and oat products) and amino acids (fish, egg, milk, and beans) in the Nordic area is 26.7 g for each individual, or around 22% of the total protein consumption. Most cereal protein comes from wheat, which contains approximately 22.1 g per day. Rye, oats, and grain provide about 2.0 g, 0.8 g, and 0.6 g of protein per person per day, respectively. Oats are a grain with unknown potential to produce nutritious, sustainable foods. Oat β-glucan has been shown to promote both gut microbiota and postprandial glucose management, leading to an increase in the production of oat products and the availability of oat meals with health-supporting qualities in several basic food categories [37]. Despite a long heritage of eating oat- and rye-based meals, as well as the rise in specific oat-based cuisines, the predominant source of cereal protein in the Nordic area remains the early stage of consumption of wheat [38]. Furthermore, oat phytochemicals, fish, green tea, berries, and broccoli have been reported to have anti-inflammatory properties, which involvethesuppression of tumor necrosis factor (TNF)-α production andinterleukin (IL)-1β [39].

### 2.3. African Heritage Diet

The African Heritage diet promotes natural components that can be potentially beneficial for African-American populations since they have much higher risks of long-term health concerns associated with their diets [40]. The study involved participants that comprised African-American individuals between the ages of 18 and 65 who were overweight or obese (body mass index: 25.9–49.9) and included participants who were non-pregnant, without type2 diabetes or suffering from untreated thyroid diseases, and the findings showed an improvement in body weight and cardiovascular diseases (CVDs) risk factor outcome provided with the African Heritage diet [41]. The African Heritage food pyramid strongly emphasizes eating a diet rich in plants, such as vegetables, fruits, grains, beans, and nuts, which reduces body weight and waist edge and reduced blood pressure [42] (Table 1). A broad network of dishes from Africa, African-Americans, the Caribbean, and South America is included in the process, instructing people regarding traditional spices, ingredients, and preparation methods [43].

### 2.4. Traditional Asian Diets

**Figure 1:**
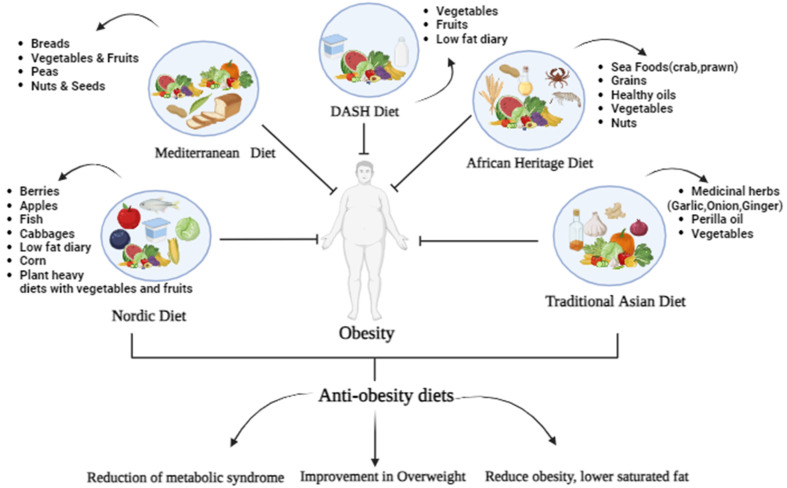
Dietary patterns of different regions and their outcomes.

The lack of potentially adequate evidence pertains to traditional regional dietary practices from other parts of the world that follow similar health-enhancing concepts. The diet of this population might be enhanced without affecting its natural composition simply by restricting the amount of grains consumed and encouraging the consumption of more nutritious fruits and vegetables as dietary sources for carbohydrates and other micronutrients [44], consisting mostly of fish and legumes in comparison to red meat, cultured cuisine, indigenous land and sea veggies, and oils made from sesame, perilla, and sesame seeds [45] (Figure 1).

The Asian diets that originate from different parts of Asia, like the Korean diet, frequently include a variety of small-portioned foods (including preventing overeating and stability into the total homeostasis of our body and are prepared within the household using seasonal ingredients; as when compared to foods cultivated regardless of the season, fruits that are picked at the peak of their initial pulpiness can offer additional health advantages) [46]. The conventional Korean diet does not contain as many fried items as the Western diet. Dietary factors, including body weight, body mass index that was calculated by weight (kg)/height (m^2^), body fat, and lean mass, were observed using a bioelectric impedance analyzer and were positively impacted by the Korean diet [45,47].

The Chinese diet comprises rice or noodles, soups, vegetables (mainly broccoli, cabbage, potato, and tomato), steamed bread or dumplings, fruits, vegetables, and soy. In contrast, seafood includes turtles, shrimp, cuttlefish, squid, shark, abalone, and meat [40,48]. The Chinese diet has the ability to reduce the risk of metabolic syndrome, obesity, hypertriglyceridemia, and hypertension [45] (Table 1). According to studies, the diet can lower BMI while maintaining the physique associated by 28% of those who were surveyed for a Western diet roughly for a year after valuation was conducted, represented by 28 in group A (*n* = 142) that provided the basic traditional Chinese diet (BCTD) and only 6 in group B (*n* = 142) that providedthe Western standard diet (WSD) [49].

The Japanese diet consists of seaweed, fruits, vegetables, and mushrooms. A Japanese tradition involves using chopsticks and switching between small-portioned items during each course, where the umami flavor found in Japanese cuisine is included to promote satiety and prevent eating excessively [50]. Some Japanese recipes are flavored with umami from konbu, resulting in tastier and healthier meals with lower fat, sugar, and salt levels. Similarly, it has been demonstrated that substituting soy sauce, which is high in umami, for salt can cut down on salt intake by up to 50% without affecting consumer approval [51].

### 2.5. Dietary Approaches to Stop Hypertension (DASH)

The DASH (Dietary Approaches to Stop Hypertension) dietary plan is an acceptable dietary pattern for people associated with diabetes. In addition to promoting blood pressure control, this dietary pattern has been shown to improve insulin resistance, hyperlipidemia, and overweight/obesity. The DASH diet analysis promotes a diet that emphasizes consuming a range of fruits, veggies, legumes, and whole-grain foods, as well as different sources of animal protein such as seafood, meat, eggs, legumes, nuts, seeds, and soy. Therefore, considering the patient’s general health and nutritional objectives, the optimal caloric intake must be evaluated. The diet followed by DASH may be personalized to meet the needs of those who want to prevent obesity [52]. Individuals who first followed a traditional diet consisting of foods that are typical of American eating habits had considerably lower blood pressure reductions than those who followed a diet rich in fruits, vegetables, low-fat dairy, and foods with lower amounts of saturated and total fat and cholesterol [53] (Table 1).

**Table 1:** Dietary patterns of different regions in maintaining obesity.

Diets	Phytonutrients	Regions	Outcomes	References
Mediterranean diet	Fruit, vegetables, breads, other forms of cereals, potatoes, beans, nuts, and seeds	Africa, Asia, and Europe	Monitoring diet, predominantly low in saturated fat; reduced amount 7–8%	[28,29,30]
Nordic diet	Apples, pears, berries, root and cruciferous vegetables, cabbages, whole grains, rye bread, intake of fish, low-fat dairy products, potatoes, and vegetable fats	Northern Europe and Europe	Stimulate low glycemic action, the contented of high glycemic index, lower effect, and blood sugar level	[33,35,36]
African Heritage diet	A plant-heavy diet with fruits, vegetables, tubers, grains, beans, nuts, healthy oils, and seafood	African, African-American, Caribbean, and South American	Reduced body weight and waist edgesand reduced blood pressure	[41,42,43]
Traditional Asian diets	Rice, whole grains, fermented food, indigenous land and sea vegetables, proteins(legumes and fish meat), medicinal herbs (garlic, green onions, andginger), sesame, and perilla oils	Asia	Lower risk of metabolic syndrome, obesity, hypertriglyceridemia, and hypertension	[44,45,48]
Dietary Approaches to Stop Hypertension (DASH)	Rich in fruits, vegetables, and low-fat dairy	East Asia	Developments to lower overweight/obesity, low-density lipoprotein cholesterol (LDL-C), and total cholesterol	[52,53]

## 3. Bioavailability of Phytonutrients in the Human Metabolism System

Several key factors influence the bioavailability of phytonutrients in the human metabolism system. During digestion, phytonutrients undergo enzymatic breakdown in the gastrointestinal tract before being absorbed through the intestinal wall and entering the bloodstream [54,55]. However, digestion efficiency can vary depending on the specific phytonutrient and an individual’s digestive enzymes.

The presence of other substances in the diet, such as dietary fibers or fats, can interact with phytonutrients and impact their absorption [56,57,58]. For example, dietary fat can enhance the absorption of fat-soluble phytonutrients like carotenoids. Moreover, individual differences, including factors like age [59], genetics [60], gut health [61], and specific health conditions, contribute to variations in phytonutrient bioavailability among individuals. Genetic variations, for instance, can influence the metabolism and absorption of phytonutrients [62].

Furthermore, the synergistic effects of phytonutrients with other compounds in whole foods are crucial for enhancing their bioavailability [63,64,65]. Consuming whole foods rather than phytonutrient supplements is generally recommended to maximize the absorption and bioavailability of phytonutrients, as certain nutrients or compounds in whole foods can enhance their absorption [66].

The processing and preparation methods of plant-based foods also impact the bioavailability of phytonutrients. Factors such as heat, light, and oxidation can lead to the degradation of some phytonutrients. However, certain cooking techniques like steaming can improve the release and absorption of phytonutrients, enhancing their bioavailability [67].

Thus, understanding these key factors is vital for optimizing the bioavailability of phytonutrients and designing dietary strategies that maximize their potential health benefits. By considering digestion and absorption processes, interactions with other substances, the role of the gut microbiota, individual differences, and the effects of cooking and food processing, individuals can make informed choices to enhance the bioavailability of phytonutrients and support overall well-being.

### 3.1. Digestion and Absorption

Phytonutrients, bioactive compounds derived from plant-based foods, have gained significant attention due to their potential health benefits. However, the bioavailability of phytonutrients, primarily influenced by the efficiency of their digestion and subsequent absorption in the gastrointestinal (GI) tract, plays a crucial role in determining their physiological effects [54]. Upon consumption, phytonutrients enter the GI tract, where various conditions influence their fate. Digestion begins in the oral cavity, where mechanical breakdown and enzymatic action initiate. Once in the stomach, the acidic environment can further facilitate the breakdown of certain phytonutrients [68,69]. However, it is within the small intestine where most phytonutrient digestion occurs. Enzymes, including proteases, lipases, and carbohydrases, act on different classes of phytonutrients, breaking them down into smaller, more absorbable compounds [70].

The efficiency of phytonutrient digestion can vary depending on multiple factors. Firstly, the specific phytonutrient structure plays a role. Some phytonutrients possess complex chemical structures, such as glycosides or conjugated forms, requiring specific enzymes for hydrolysis. The availability and activity of these enzymes vary among individuals, contributing to inter-individual variability in digestion efficiency [71,72,73,74].

Secondly, individual differences in digestive enzyme production and activity can significantly influence phytonutrient digestion. Genetic variations, age, and overall gut health can impact digestive enzymes’ levels and functionality, affecting the breakdown of phytonutrients [55,75]. For instance, lactase deficiency can hinder the digestion of lactose-containing phytonutrients [76].

Once phytonutrients are digested into smaller components, they are absorbed into the bloodstream through the intestinal wall [71]. The absorption process primarily occurs in the small intestine, where various mechanisms are involved [71]. Passive diffusion is the primary mode of absorption for lipophilic phytonutrients, while hydrophilic compounds often require specific transporters for absorption [77,78].

### 3.2. Interaction with Other Substances

One important aspect is the interaction of phytonutrients with substances such as dietary fibers or fats, which can have both positive and negative effects on their absorption and subsequent physiological effects absorption [56,57,58,79]. Understanding these interactions is crucial for optimizing phytonutrients’ bioavailability and health benefits.

Dietary fibers, present abundantly in fruits, vegetables, and whole grains, can interact with phytonutrients in several ways [63,80]. Firstly, fibers can physically trap phytonutrients, forming complexes that may hinder their absorption [81,82]. However, in some cases, certain types of fibers, particularly soluble fibers, can positively impact phytonutrient bioavailability [83,84]. Soluble fibers can form gel-like structures in the gut, creating a conducive environment for absorbing phytonutrients [85,86].

Dietary fats can also significantly affect the bioavailability of certain phytonutrients, particularly fat-soluble ones [87]. For example, carotenoids, a class of phytonutrients abundant in fruits and vegetables, are better absorbed in dietary fat [88]. Fats can enhance the solubilization of lipophilic phytonutrients, facilitating their incorporation into mixed micelles and subsequent absorption through the intestinal wall [89,90]. Thus, consuming plant-based foods and a source of dietary fat, such as olive oil or avocado, can optimize the absorption and bioavailability of fat-soluble phytonutrients.

Moreover, the specific food matrix in which phytonutrients are found can impact their interaction with other substances and subsequent bioavailability [54]. Whole foods contain a complex mixture of phytonutrients, fibers, fats, proteins, and other components that can act synergistically or competitively. Certain nutrients or compounds in the food matrix can enhance the absorption of phytonutrients through mechanisms such as increased solubility, improved stability, or the facilitation of transport processes [82,91].

### 3.3. Individual Differences

The bioavailability of phytonutrients, the extent to which they are absorbed and utilized by the body, can exhibit significant inter-individual variation. This variation arises from several factors, including age, genetics, and specific health conditions, which can impact the metabolism and absorption of phytonutrients [92,93].

Genetic variations among individuals can significantly influence the bioavailability of phytonutrients. Genetic polymorphisms in enzymes responsible for phytonutrient metabolism and absorption can affect their efficiency and contribute to individual differences [62,94]. For example, variations in genes encoding phase I and phase II enzymes, such as cytochrome P450s and glucuronosyltransferases, can influence the metabolism and clearance of phytonutrients [95,96,97]. These genetic differences can result in variations in bioavailability and potentially alter the health effects associated with phytonutrient consumption.

Age is another important factor that influences phytonutrient bioavailability. Infants, children, and older people may exhibit differences in the absorption and metabolism of phytonutrients compared to adults [98]. Age-related changes in digestive enzyme production, gastrointestinal physiology, and gut microbiota composition can impact the bioavailability of phytonutrients [72,75,99].

The presence of specific health conditions can also impact the bioavailability of phytonutrients. For example, individuals with malabsorptive disorders, such as celiac disease [100,101,102] or pancreatic insufficiency [103,104], may experience reduced absorption of phytonutrients. Additionally, certain chronic diseases or conditions, such as liver disease [105,106] or kidney dysfunction [105,106], can affect the metabolism and clearance of phytonutrients, altering their bioavailability. Moreover, the use of medications, such as proton pump inhibitors [105,106] or certain antibiotics [105,106], can interfere with the absorption and metabolism of phytonutrients, further contributing to inter-individual differences in bioavailability.

### 3.4. Cooking and Food Processing

The processing and preparation of plant-based foods can significantly impact the bioavailability of phytonutrients [54]. Various factors, including heat [57], light exposure [107], and oxidation [108], can influence the stability and availability of these compounds. Understanding the effects of different cooking and processing methods is crucial for optimizing the diet’s retention and bioaccessibility of phytonutrients.

One important consideration in cooking plant-based foods is the potential for the heat sensitivity of phytonutrients. Some phytonutrients, such as vitamin C [109] and certain water-soluble compounds [110], are susceptible to degradation when exposed to high temperatures. Prolonged cooking or boiling can lead to the leaching and loss of these heat-sensitive phytonutrients [111]. Therefore, methods like steaming or stirfrying, that involve shorter cooking times and minimal contact with water, are often preferred to preserve the bioavailability of these compounds [112].

Light exposure can also impact the stability and bioavailability of phytonutrients. Some compounds, such as carotenoids [112] and flavonoids [113], are prone to degradation when exposed to light. Therefore, storage and handling practices that minimize light exposure, such as storing fruits and vegetables in opaque containers or wrapping them in protective coverings, can help preserve the integrity of light-sensitive phytonutrients and maintain their bioavailability [114,115].

Oxidation is another important factor to consider in the processing and preparation of plant-based foods. Oxidative processes can degrade phytonutrients and reduce their bioavailability [116]. For example, phenolic compounds widely distributed in plant-based foods are susceptible to oxidation [117,118]. Cutting, peeling, or chopping fruits and vegetables can expose these compounds to oxygen, leading to enzymatic or chemical reactions that result in their degradation [119]. Minimizing exposure to air [120] and using antioxidant-rich ingredients like citrus juices [121] or herbs/spices [122] with high antioxidant content can help mitigate oxidation and preserve phytonutrient bioavailability.

### 3.5. Bioavailability of Phytonutrients as Anti-Obesity Activities

Scientific studies have provided evidence regarding the bioavailability of phytonutrients with anti-obesity activities, shedding light on their potential role in combating obesity. Several phytonutrients have demonstrated anti-obesity activities, such as polyphenols [123,124,125,126], flavonoids [127,128,129,130], and carotenoids [131,132,133]. These compounds have been shown to regulate various mechanisms involved in energy balance, adipose tissue metabolism, and inflammation, all of which are critical factors in the development of obesity. However, the bioavailability of these phytonutrients is a crucial consideration for their functional efficacy in obesity prevention or treatment [134,135].

The functional bioavailability of phytonutrients for obesity depends not solely on their absorption but also on their interactions with target tissues [136] and their metabolic fate [137]. After absorption, phytonutrients can undergo biotransformation by enzymes, including those in the gut microbiota, leading to the formation of metabolites with distinct biological activities [93]. These metabolites can directly affect adipose tissue metabolism, energy expenditure, and inflammation, contributing to the anti-obesity properties of phytonutrients [138,139].

## 4. Phytonutrients with Anti-Obesity Effects

There are several anti-obesity drugs in the market; however, their usage has been limited due to their adverse side effects. Anti-obesogenic medications currently on the market fall mostly into two categories. The first is orlistat, which blocks pancreatic lipase activity to reduce intestinal fat absorption, and the second is sibutramine, an anorectic or appetite suppressant. These therapies are pricey and prohibitive, particularly for those in developing nations, and have been associated with adverse health outcomes like increased blood pressure, dry mouth, constipation, headaches, and insomnia [140]. Hence, due to such adverse effects, dietary therapies utilizing naturally occurring bioactive food components have become attractive therapeutic options for treating obesity and metabolic illnesses. Therefore, in recent years, researchers have concentrated on food, especially from plants and animals, in search of components that are efficient and beneficial for lowering obesity and other related problems [141]. These phytonutrients showed potential anti-obesity and anti-diabetes effects by altering the following physiological pathways such as controlling hunger, metabolism, and insulin sensitivity. Consuming phytonutrients is considered a secure, readily accessible, and affordable method of controlling diabetes and obesity [7]. Phytoconstituents possess five potential mechanisms of action to fight against obesity, which include lipase inhibitors, appetite suppressants, thermogenic energy expenditure regulators, adipocyte differentiation regulators, and lipid metabolism regulators [142].

**Figure 2:**
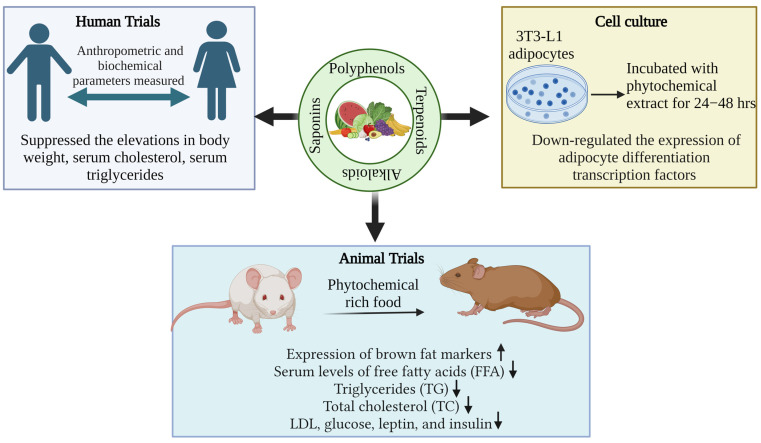
Methodology adapted for testing anti-obesity effects of phytochemicals.

Among phytochemicals, polyphenols, tannins, flavonoids, terpenoids, saponins, alkaloids, steroids, glycosides, and proteins are found in plants, and their respective products are crucial for the treatment of various health problems [142,143]. Polyphenols and flavonoids like resveratrol, quercetin, kaempferol, myricetin, catechins, cyaniding, and anthocyanin [144,145,146] and alkaloids like caffeine, capsaicin, and ephedrine were reported with effective anti-obesity effects via both increased lipolysis as well as thermogenesis along with reduced appetite [147]. Similarly, terpenoids like β-carotenoids, cryptoxanthin, fucoxanthin, zeaxanthin, and lycopene [148] and saponins like ginsenosides [149] showed the potential anti-obesity effect, which is briefly described below. Figure 2 depicts the methodologies adapted for assessing the anti-obesity impact of phytochemicals.

### 4.1. Polyphenols

As secondary metabolites, polyphenols constitute a large bioactive substance naturally found in plants, and their presence in diets may help in the beneficial modulation of various factors related to health variables that are related to obesity by consuming dietary polyphenols [124]. Several mechanisms (either an individual or in combination effect) for polyphenols have been reported regarding their anti-obesity functions via inhibiting enzymes and adipocyte differentiation, regulation of lipid metabolism, suppressing appetite or stimulation of energy expenditure, and modulation of microbiota in the gut [150]. The in vitro experiment on catechin from green tea extract (GTE) by Weng et al. [151] showed reduced lipid accumulation by preventing differentiating 3T3-L1 preadipocytes into adipocytes and increased the turning of white adipocytes into brown due to the presence of (-)-Epigallocatechin gallate EGCG. Authors suggested that EGCG could enhance glucose homeostasis in the adipocyte cell line 3T3-L1 by restoring the balance of redox factors and addressing mitochondrial dysfunction. Further, it was also demonstrated that taking GTE at the dose of 583 mg of catechins in a daily diet for 12 weeks caused reductions in the mass of adipose tissue, body weight, and serum levels of LDL-C.Also, GTE supplementation ameliorates lipid accumulation via inhibition of 3T3-L1 adipocytes following conversion of white adipocytes into brown [151]. In another study, Tung et al. [152] studied the browning of 3T3-L1 adipocytes after administration of 20 mM curcumin, thereby raising the expression of brown fat markers (PGC-1, PPAR, UCP1 PRDM16 C/EBP, Tmem26, Cidea, and FGF21Tbx1) and demonstrating that curcumin changes white adipocytes into beige adipocytes. Curcumin reduced TG concentration in brown-like adipocytes by raising mitochondrial CPT-1 and cytochrome C protein levels, which increased fat oxidation. Curcumin also raised pACC, pAMPK/AMPK ratio, and HSL expression to promote lipolysis and inhibit FA formation, respectively [152].

In 2015, Alkhalidy et al. [153] used kaempferol (0.05% in the diet) to enhance skeletal muscle glycolysis, glucose absorption, glycogen synthesis, AMPK activity, and Glut4 expression on the cellular and molecular levels. In addition, adding kaempferol to the diet considerably reduced hyperglycemia in old, obese diabetic mice and retained functional islet mass. These findings suggest that the phytonutrient kaempferol may be taken as a dietary supplement to avoid metabolic diseases linked to aging and obesity [153]. At the cellular level, AMPK restricts gluconeogenesis, regulates mitochondrial biogenesis, and promotes fatty acid oxidation. Similarly, supplementation of MGP (muscadine grape) and MWP (muscadine grape wine) has phytochemical compositions quercetin, myricetin, kaempferol, ellagic acid, cyanidin 3,5-diglucoside, delphinidin 3,5-diglucoside, peonidin 3,5-diglucoside, malvidin 3,5-diglucoside,andpetunidin/pelargonidin 3,5-diglucoside and total anthocyanins feed in the HF diet of mice, showing significantly reduced fat mass by 29 for MGP and 12.5%, for MWP, as compared to the HF group [154]. Compared to mice fed the HF diet alone, the addition of MGP and MWP reduced the liver’s weight by 41.6% and 37.5%, respectively. In contrast, the weight difference between lean and obese mice’s hepatic tissues was 54% [154]. Supplementation of raspberry juice and puree concentrates (RJC and RPC), along with the mixture of ellagic acid (EA) and raspberry ketone (RK), when added to high-fat diets of mice, considerably reduced the amount of body weight increase [155]. Recently, Xu et al. [156] used green tea (*Camellia sinensis*, Theaceae) extract at diet doses of 400 or 800 mg/kg and reported anti-obesity effects on rats with high-fatdiets for six weeks. The findings showed that green tea extract possesses a suppressive effect on body weight gain and fat buildup due to polyphenols and polysaccharides. Polyphenols, such as caffeine, and polysaccharides enhance the antioxidants and blood lipid levels, reduce serum leptin levels, prevent fatty acid absorption, and lower IL-6 and TNF-gene expression levels. Also, it was demonstrated that polysaccharides and polyphenols collaboratively reduce blood leptin levels with anti-inflammatory effects [156].

**Figure 3:**
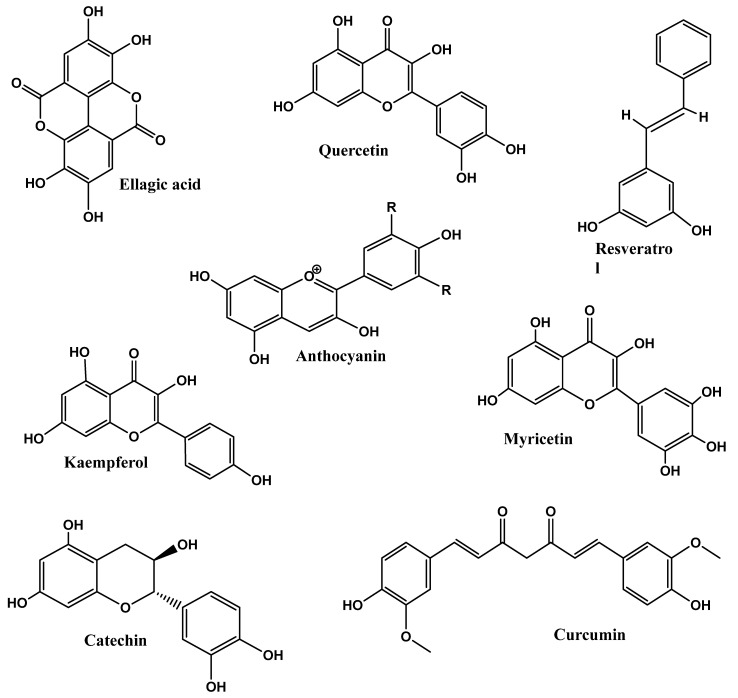

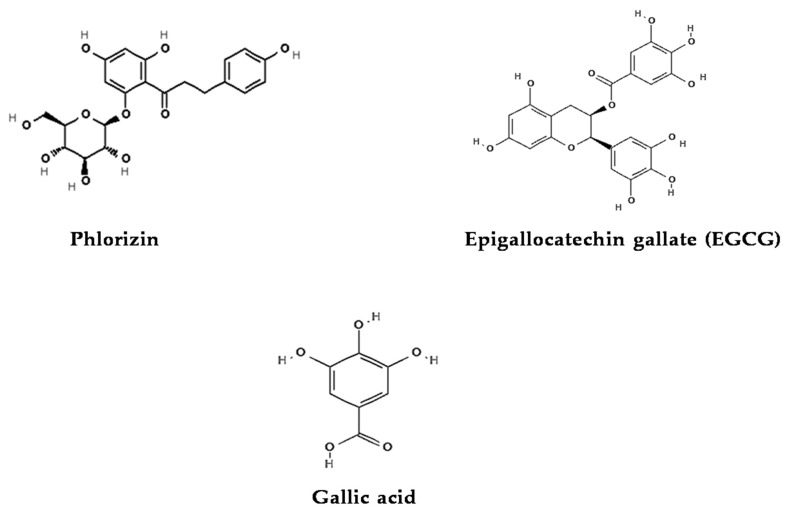
Chemical structures of phytonutrient potential as anti-obesity.

In clinical trials performed by Suzuki et al. [157], GTE (green tea extract) enriched (280 and 360 g GTE) in rye bread was used in randomized single-blinded studies for both men and women. They provided 242.1 and 188.3 mg of EGCG, respectively. Compared to the control, GTE-enriched bread consumption caused a significant reduction in waist circumference, i.e., −1.22 cm and was beneficial in maintaining low blood pressure [157]. Figure 3 depicts the chemical structures of polyphenol’s potential as anti-obesity.

#### 4.1.1. Phlorizin

Phlorizin (PHZ) is a nutrient found in apples that helps to make one healthier. The amalgamation of PHZ and other strategies aided in curbing weight gain attributed to overconsumption, ultimately resulting in reduced body mass. The outcome of combining PHZ was not due to reduced food intake or energy consumption. The decrease in accumulated fat provided evidence that this combination might result in its better effectiveness [158] (Figure 3). The gut lining’s impairment, which results from excessive intake of fatty food, is mended by the body’s generation of glucagon-like peptide (GLP-2). Overconsumption of fatty foods alters favorable bacteria in the gastrointestinal microbiome. PHZ can help fight obesity by focusing on the relationship between gut bacteria and gut lining [158]. An in vitro study isolated human plasma where the activity was observed bytheinhibition of LDL oxidation at physiological levels, LDL-cholesterol oxidation [159,160]. Phlorizin during in vivo studies was reported to prevent weight gain in mice while their food consumption remained constant. The reduction in body weight corresponded with a decrease in the number of abdominal fats [161]. The PHZ group had less fat and smaller adipose cells than the high-fat diet (HFD) group. The PHZ group exhibited improved lipid levels in both their blood and liver. Individuals with PHZ who exhibited lower levels of certain enzymes involved in fat production, storage, and cholesterol synthesis also showed an evident reduction in liver fat [49,162].

#### 4.1.2. Epigallocatechin Gallate (EGCG)

The consumption of energy enhancement via the increased process of thermogenesis where obesity was caused in male C57BL/6J mice and the phytonutrient epigallocatechin gallate is observed to have the ability to cause changes in blood sugar and triglyceride levels significantly by decreasing/lowering lipid formation in adipose tissues and also by reducing body fat growth [163]. The primary catechin in green tea, (-)-Epigallocatechin-3-gallate (EGCG), offers several possible health benefits, notably a reduction in body weight and possibly adipose tissue weight. The stimulation of AMPK in the liver, skeletal muscle, and white adipose tissue, as well as a decline in caloric intake, are proposed as possible methods by which EGCG may lower body mass index. Epididymal adipose tissue weight and blood lipid properties, such as triglyceride, cholesterol (CHOL), and high- and low-density lipoprotein CHOL (HDL-C, LDL-C) levels, were all significantly impacted by EGCG [164]. The potential of EGCG to regulate body fat content was demonstrated in an in vitro investigation using *Escherichia coli* and *C. elegans* strains with the OP50 diet, where the prevention of adipogenesis led to a decrease in the fat content in *C. elegans*. This was demonstrated by the lowered ATGL-1 gene expression level following EGCG administration [160]. In overweight mice affected by a dietary regimen, EGCG was found to provide benefits in decreasing body fat through reduced calorie intake, enhancing both in vivo and in vitro oxidation of fatty acids and lipolysis [164]. In a clinical trial where 15 women with central obesity were screened to regulate plasma cholesterol and triglycerides, 102 of them with a body mass index (BMI) ≥ 27 kg/m^2^ and a waist circumference (WC) ≥ 80 cm were observed to be able to decrease body weight and BMI in obese women after a 12-week treatment, alsowitha significant reduction in waist circumference [165] (Figure 3).

#### 4.1.3. Gallic Acid

Gallic acid (GA) is a naturally occurring physiological phenolic acid with a chemical composition of 3-, 4-, and 5-trihydroxy benzoic acid. It can be extracted from vegetables and fruits and is thus prevalent in plant products, such as green tea and fruit juices, which are good for maintaining energy and homeostasis [166]. The ability of gallic acid could initiate an alteration by inhibiting lipogenesis and lowering fat accumulation in obese people. The process of synthesizing fat in our system from components like carbohydrates is called lipogenesis [167]. GA changes the inter-scapular brown adipose tissue’s thermogenic genes and activates the Adenosine 5′-monophosphate-activated protein kinase/Sirtuin 1/Peroxisome proliferator-activated receptor γ coactivator-1 (AMPK/SIRT1/PGC-1) pathway to carry out its favorable metabolic activities [168]. In the in vitro study, the size of adipocytes, because adipocyte hypertrophy is associated with adipose tissue inflammation and metabolic disorder Murine 3T3-L1 preadipocytes and RAW 264 macrophages average fat cell18 size in WAT, was significantly lower in the GA group compared to the control group [169]. In the in vivo reported studies, for the obesity in male C57BL/6 mice, there were formed adipose and fat contents that provided a drastic reduction in lipolysis that was induced, while Fas ligand (FAS), which is a cell surface death receptor, was suppressed to prevent lipogenesis, controlling the degradation of lipids and decreasing fatty tissue and calories [170]. Obese human subjects receiving capsules containing 200 mg of gallic acid and 50 mg of a Chinese herbal decoction three times a day for 24 weeks did not experience weight loss or a decrease in food intake in humans, principally due to the inability to achieve adequate serum levels [167] (Figure 3).

#### 4.1.4. Resveratrol and Quercetin

Quercetin exerts anti-obesity activity via the mitogen-activated protein kinase (MAPK) and 5′-adenine monophosphate-activated protein kinase α1 (AMPKα1) signaling pathways [171]. Resveratrol, a phytoalexin originating from the skin and seeds of grapes and red wine, may also protect against diet-induced obesity in vivo studies and metabolic diseases, including hepatic steatosis and insulin resistance [172]. The anti-obese effect of the combination of resveratrol and quercetin (CQR) is related to a reduction in body weight gain, adipocyte diameter, and adipose tissue weight and an improvement in dyslipidemia in serum. Its anti-obese effect is closely related to its anti-inflammatory properties by which it regulates adipokine release and initiates the AMP-activated protein kinase/Sirtuin 1 (AMPKα1/SIRT1) signaling pathway. These effects indicated that CQR can potentially reduce HFD-induced obesity and inflammation [173]. Inan invitro study in human SGBS adipocytes, it led to the inhibition of adipogenesis by decreasing gene expression levels of the key adipogenic factors peroxisome proliferator-activated receptor γ (PPARγ) and CCAAT/enhancer binding protein α (C/EBPα) and reducing levels of adipokines (ANGPTL4), adipsin, and PAI-1 as well as of glycolysis-associated enzymes ENO2, PFKP, and PFKFB4, all of which are associated with obesity and adipose tissue dysfunction [173]. In the experimental model, male Wistar rats induced obesity where the modulation of gut microbiota was observed to decrease body weight gained significantly, visceral adipose tissue weight, and adipocyte sizes [174]. A clinical trial was conducted in 11 obese, otherwise healthy men by down-regulating genes involved in intercellular junctions, Wnt signaling, angiogenesis, G protein-coupled receptors, and Notch signaling, up-regulating pathways involved in cell cycle regulation, and reducing adipocyte size, thereby, enhancing and improving adipogenesis [175,176] (Figure 3).

### 4.2. Alkaloids

Another class of bioactive compounds is alkaloids, which are low-molecular-weight and nitrogen-containing compounds. They are heterocyclic rings, which are alkaline due to the presence of a nitrogen atom [177]. These metabolites are divided into various sub-classes based on their precursor (e.g., tryptophan-derived indole alkaloids) and other classes such as piperidine alkaloids, pyrrolidine alkaloids, pyridine alkaloids, tropane alkaloids, quinolizidine alkaloids, etc. [177]. Reserpine from *Rauwolfia vomitoria*, an alkaloid, reduces blood pressure and treats hypertension by targeting blood vessels. It may also be helpful to boost metabolism and induce the excretion of fluids in obese patients. Alkaloids have anti-obesity effects due to increasing lipolysis and thermogenesis and suppressing hunger [178]. The impact of piperine on adipocyte cell line 3T3-L1 has been demonstrated to block differentiation by down-regulating PPAR, SREPB-1c, and C/EBP, suggesting that it may be useful in the treatment of metabolic disorders [179]. Relatedly, 13-methyl berberine was discovered to have the most powerful anti-adipogenic effects among the 11 protoberberines and two benzophenanthridine alkaloids by Chow et al. [180], who tested them on 3T3-L1 adipocytes. Both PPAR- and CCAAT-enhanced bindings of C/EBP were down-regulated and targeted for gene regulation. The reduction in PPAR, C/EBP, and sterol regulatory element binding protein 1 (SREBP-1) protein levels, as well as the attenuation of this lipid-reducing action by an AMP-activated protein kinase (AMPK) inhibitor followed. It further suggested that this drug’s effects depend on the AMPK signaling pathway. Previously, Ma et al. [181] isolated four main aporphine alkaloids, including 2-hydroxy-1-methoxyaporphine, pronuciferine, nuciferine, and roemerine, were isolated from the leaves of *Nelumbo nucifera*. These alkaloids exhibited concentration-dependent cytotoxicity in 3T3-L1 cells, and 2-hydroxy-1-methoxyaporphine and pronuciferine significantly increased glucose uptake in differentiated adipocytes. These findings might help explain why lotus leaves are so widely used in China for blood sugar regulation and weight loss [181]. A different component in *N. nucifera* leaves has a variety of anti-obesity effects. In the investigation by Ahn et al. [182], benzoisoquinoline alkaloids prevent the absorption and storage of fat. While flavonoids prevent fat storage, other alkaloids and metastigmanes are better at preventing fat absorption. As a result, *N. nucifera* leaves may be used to treat obesity since they control pancreatic lipase and adipocyte differentiation [182].

**Figure 4:**
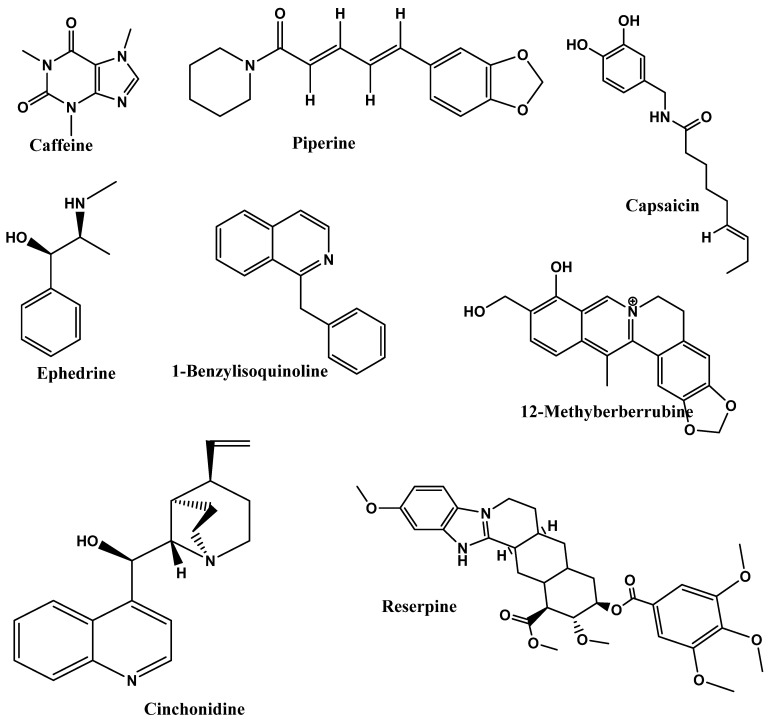
Chemical structures of potential of alkaloids as anti-obesity.

In the study by Gurung and De [183], authors compared the curcumin and EGCG (epigallocatechin gallate) with the strong alkaloids in cinchona bark, cinchonine demonstrated a higher final body weight reduction rate, and results showed that cinchonine significantly reduced plasma glucose, LDL-+VLDL-cholesterol, HDL-cholesterol, and TG levels in response to the HFD (high-fat diet)-induced hyperlipidemia and hyperglycemia in mice. Cinchonine impacts hyperlipidemia and hyperglycemia caused by an HFD, which is an early sign of metabolic syndrome and other related illnesses. In conclusion, it was suggested that cinchonine significantly reduces adipogenesis by suppressing the WNT and galanin-mediated signaling pathways. This reduces adipose tissue’s inflammation by suppressing the TLR-2- and TLR-4-mediated pro-inflammatory signaling pathways [183]. In another study by Huang et al. [184], lansiumamide B, a new alkaloid discovered in the *C. lansium* seeds, demonstrated promising anti-obesity effects in rats given an HFD, which could be used as an anti-obesity medication. According to q-RT-PCR analysis results, HFD treatment increased the expression of adipokines, leptin, and the lipogenic markers FAS (fatty acid synthase) and SREBP-1c but not the adipogenic markers AP2 (adipocyte protein 2) and PPAR2, and also lansiumamide B treatment significantly decreased the expressions of leptin, FAS, and SREBP-1c [184]. Mahanimbine, an essential carbazole alkaloid found in *Murraya koenigii* (curry leaves), prevented HFD-induced hyperlipidemia alongside fat accumulation in adipose tissue and the liver as well as the development of systemic inflammation and oxidative stress. It has been found to enhance the glucose clearance and increase the expression of insulin-responsive genes in the liver and adipose tissue when given daily along with HFD feeding for 12 weeks at both low and high doses, i.e., 2 mg/kg and 4 mg/kg body weight [185]. Ohara et al. [35] studied the effect of the consumption of a G-hesperidin (500 mg)- and caffeine (25, 50, or 75 mg)-based diet for 12 weeks on 75 healthy individuals with moderate BMIs and serum triglycerides. The result of the double-blind randomized design showedareduced abdominal fat area in G-hesperidin with 50 mg caffeine group and G-hesperidin with 75 mg caffeine group. This study suggested that combining both G-hesperidin (500 mg) and caffeine (50 and 75 mg) could be beneficial for treating obesity. Figure 4 represents the chemical structures of the potential of alkaloids as anti-obesity.

### 4.3. Terpenoids

**Figure 5:**
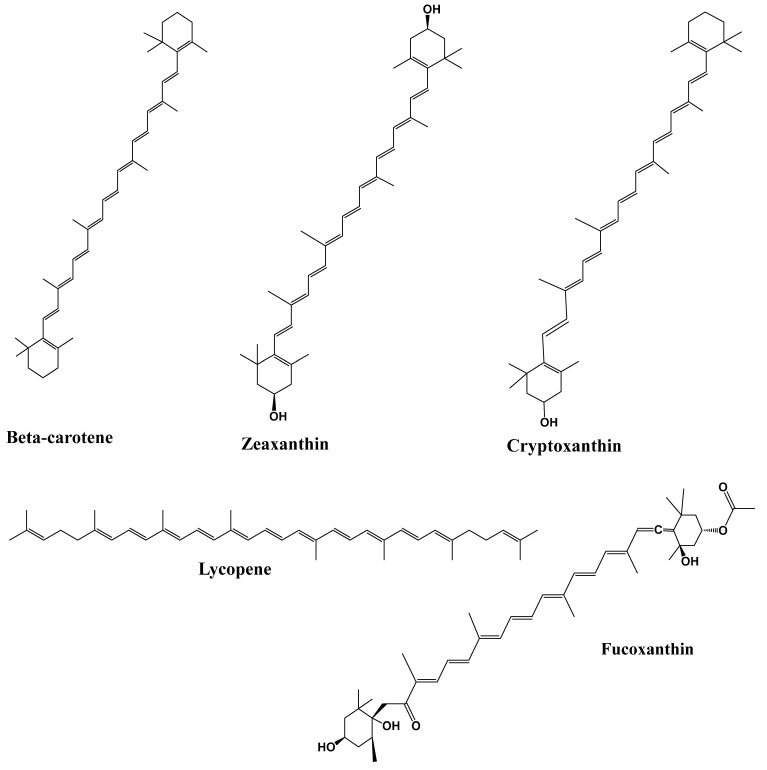
Chemical structures of potential of terpenoids as anti-obesity.

The largest secondary metabolites in nature are terpenes (plants, fungi, marine organisms, and animals). Terpenes are mostly found in essential oils as a major component. It comprises isoprene units with five carbon and eight hydrogen atoms and is regarded as the basic unit of all terpene kinds. There are two isoprene units in monoterpene (C10), three in sesquiterpenes (C15), four in diterpenes (C20), five in sesterpenes (C25), six in triterpenes (C30), and eight in tetraterpenes (C40) [186]. An important site for carotenoids and retinol storage is adipose tissue. According to estimates, 15–20% of the body’s total retinol is stored in the WAT of the rats, primarily in the adipocytes as non-esterified retinol [187]. Adipocytes contain carotenoids mostly connected with triacylglycerol in the lipid droplet and cell membranes. Thus, several reports examined the role of carotenoids in adipogenesis, which may aid in controlling obesity by limiting the buildup of lipids in adipocytes. Most of the observed effects are interfered with nuclear receptors like RAR, RXR, or PPAR to suppress adipocyte development. In addition to producingapo-140-carotenal and suppressing PPAR, PPAR, and RXR activation, -carotene also suppressed adipogenesis by producing all-trans retinoic acid [148]. Adipose tissue, liver, kidney, pancreas, brain, ovaries, gut, and eyes are just a few of the organs and/or tissues in which lycopene demonstrates anti-obesity properties. According to epidemiological research, eating foods high in lycopene may help reduce the chance of developing obesity. Lycopene’s cis-isomers mostly distribute in the liver and adipose tissues and are more accessible and better absorbed than trans-lycopene. In brown adipose tissue (BAT), lycopene can enhance the browning of white adipose tissue (WAT), elevate the expressions of thermogenic genes (UCPs), reduce the expression of fibroblast growth factor-21 (FGF21), and increase the expressions of PPAR, SIRT1, and UCP1 [188]. A xanthophyll with a distinctive structure called fucoxanthin has received much interest for its ability to fight against obesity. An increase in Adrb3 mRNA expression in mice’s WAT, which may be responsible for adaptive thermogenesis via SNS activation and UCP1 expression, may cause the potential anti-obesity benefits of a fucoxanthin diet. Therefore, carotenoid has been proposed as the primary pharmacophore of anti-obesity activity [189]. Zeaxanthin is a form of carotenoid that has been shown to have anti-lipogenesis properties [190]. It has also been shown to impact obesity in C57BL/6J mice fed a high-fat diet by preventing adipocyte 3T3-L1 cell adipogenesis. Zeaxanthin considerably reduced the intracellular lipid content of adipocytes in a dose-dependent manner. Zeaxanthin administered orally at a dose of 20 mg/kg slowed the development of obesity and enhanced dyslipidemia in mice with obesity brought on by a high-fat diet. In vitro and in vivo, it reduces the transcriptional factors and adipocyte-specific genes involved in adipogenesis, demonstrating an anti-adipogenic action. The MDI (0.5 mM 3-isobutyl-1-methylxanthine, 1.0 mM dexamethasone, and 1.0 g mL^−1^ insulin) and HFD (high-fat diet)induced suppression of AMPK phosphorylation in both adipocytes and epididymal adipose tissues, respectively, via zeaxanthin treatment, which subsequently changed the energy metabolism. These findings confirm that zeaxanthin inhibits lipogenesis, induces AMPK activation, and reduces intracellular lipid content, adipocyte size, and adipose mass [190]. In the previous study, consumption of 90 g pre-meal raw and ripe tomatoes in young women was observed for 4 weeks daily before lunch [191], and biochemical and anthropometric parameters were measured. The study revealed that after 4 weeks, significant reductions in %fat, triglycerides, body weight, fasting blood glucose, uric acid, and cholesterol in young adult women were observed. Tomato in pre-meal is assumed to have high lycopene content, which is responsible for hypolipidemic, hypouricemic, and hypoglycemic effects in young women. Figure 5 represents the chemical structures of the potential of terpenoids as anti-obesity.

### 4.4. Saponins

Many saponins are triterpene glycosides with steroids or triterpenes in the aglycone. Saponin molecules have a variety of lipophilic and lipophobic aglycone moieties that provide emulsifying properties [192]. The most widely recognized saponins are Panax ginseng, Panax japonicas, and Platycodi radix, which have been proven effective in several models to prevent or reduce obesity [193]. Saponin’s glycemic effects appear to be the result of various processes, including the improvement of the elevation of plasma insulin levels, the insulin response, and the stimulation of the pancreas via the production of insulin. For instance, it was demonstrated that saponin platyconic acid, which was isolated from Platycodi radix, promoted insulin-stimulated glucose uptake in 3T3-L1 adipocytes. In contrast, arjunolic acid, discovered in *Terminalia arjuna* Wight and Am. and other species, had an inhibitory effect on both amylase and glucosidase [194]. The macrophage-conditioned medium (RAW-CM) stimulates adipocytes, red ginseng’s saponin fraction (SF) controls the expression of adipokines, and the expression of adiponectin was elevated (more than two folds). However, its resistance expression was down-regulated (by 40%). SF considerably reduced both MCP-1 by 37% and IL-6 by 25% production in the contact system of adipocytes and macrophages. Also, in another system, the Transwell system, anSF at 100 g/mL showed a dramatically elevated quantity of hemoxygenase-1 (HO-1) by 1.5–3.5 folds and nuclear factor (-derived 2)-like 2 (Nrf2) by 2.8–3.6 folds, by enhancing Nrf2 translocation into the nucleus. Nevertheless, the Nrf2 or HO-1 knockdown condition abrogated the SF-mediated inhibitory effect on producing IL-6 and MCP-1 cytokines. This finding demonstrated the requirement for Nrf2 activation for SF-mediated prevention of obesity-induced inflammation [195].

**Figure 6:**
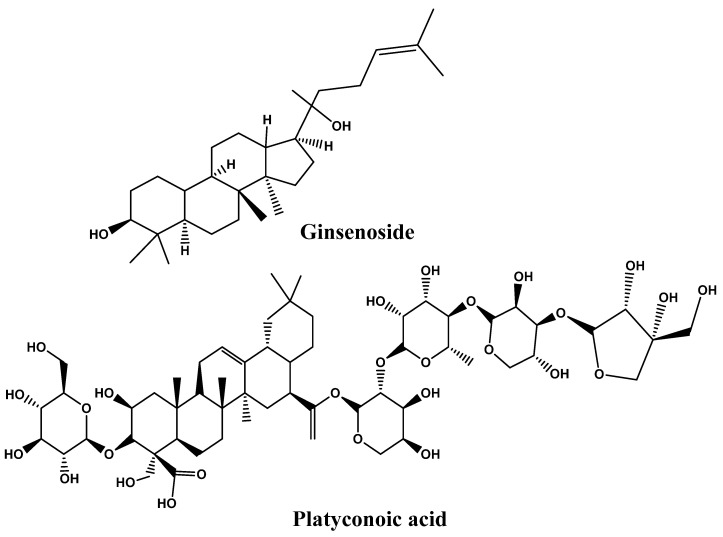
Chemical structures of potential of saponins as anti-obesity.

Findings demonstrate that the stem and leaves of *Panax ginseng* (SLG) showed significant anti-obesity effects in diet-induced obese mice, as evidenced by reduced serum levels of triglycerides (TGs), free fatty acids (FFAs), low-density lipoprotein (LDL)-cholesterol, total cholesterol (TC), insulin, glucose, and leptin with reduced overall body and liver weight. This was conducted using high-fat diet (HFD)-induced obesity in a mouse model. SLG triggers the up-regulation of CPT-1, PPAR, UCP2, ATGL, and HSL and the down-regulation of PPAR, FAS, CD36, and FATP2 in liver tissue. In addition, comparing the HFD group with the SLG groups, the former group had reduced levels of PPAR, AP2, and leptin mRNA and higher expressions of PPAR, PGC-1, UCP1, and UCP3 genes in adipose tissues. In conclusion, SLG is essential for mice fed an HFD to have anti-obesity effects, possibly due to control of thermogenesis, lipogenesis, and lipolysis [196]. The impacts of ginsenoside (SG) and saponin obtained from sea cucumber (SSC) on enhancing lipid metabolism in C57BL/6 mice fed with an HFD and receiving SSC for eight weeks showed that SSC significantly reduced HF-induced fat mass, lipid levels in both liver and serum, weight gain, and insulin levels in serum and glucose. SSC reduced high-fat diet-induced obesity in C57BL/6 mice primarily by slowing lipid production and speeding up lipid oxidation and glycolysis in the liver [197]. Song et al. [198] conducted a study on 10 obese Korean middle-aged women on the effect of Panax ginseng extract consumption for 8 weeks. Significant changes in body mass index and weight were reported, with slight differences in gut microbiota. The significant reduction in body weight by intake of ginseng extract confirms its beneficial effects on the obese population. However, its effectiveness depends on gut microbiota composition before ginseng intake. Figure 6 represents the chemical structures ofthe potential of saponins as anti-obesity and Table 2 represents the effect of phytonutrients on obesity.

### 4.5. Anthocyanins

**Figure 7:**
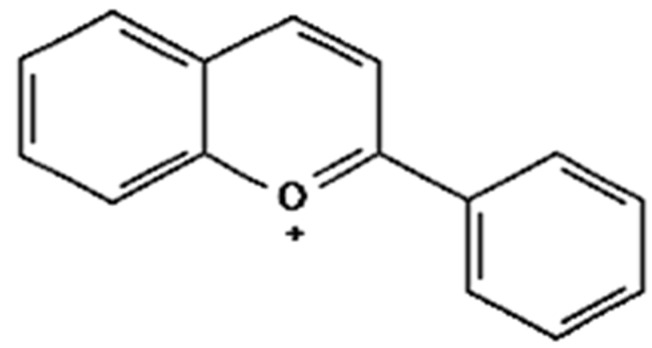
Chemical structures of potential of Anthocyanins as anti-obesity.

Anthocyanins are responsible for the red, blue, and purple colors in vegetables and are reported to possess significant anti-inflammatory properties in obese adipose tissues, whereas anti-obesity mechanisms are associated with anthocyanins [201]. In vitro studies on anthocyanins prevent nuclear factor kappa B (NF-κB) activation, thus decreasing the entire downstream cascade of pro-inflammatory mediators, such as C-reactive protein (CRP), interleukin (IL)-6, and tumor necrosis factor (TNF)-α,and improving gut dysbiosis, restoring a balanced gut microbiota. The IL-6 gene in lipopolysaccharide (LPS)-induced adipose stem cells treats obesity-related inflammation and chronic disease [202] (Figure 7). Male Sprague–Dawley rats induced obesity reduction in food intake through the regulation of neuropeptide Y and *γ*-aminobutyric acid receptor in the hypothalamus where it has been observed that there is a significant decrease in body weight gain by 15.76% and in daily food intake by 19.10%, as compared to the control [203]. In a clinical trial involving healthy male volunteers, dried purple carrots (118.5 mg/day for 4 weeks) lowered lipids, body composition, and inflammation in obese adults, eventually resulting in an overall reduction in body mass index and low-density lipoprotein cholesterol [204].

#### Cyanidin

**Figure 8:**
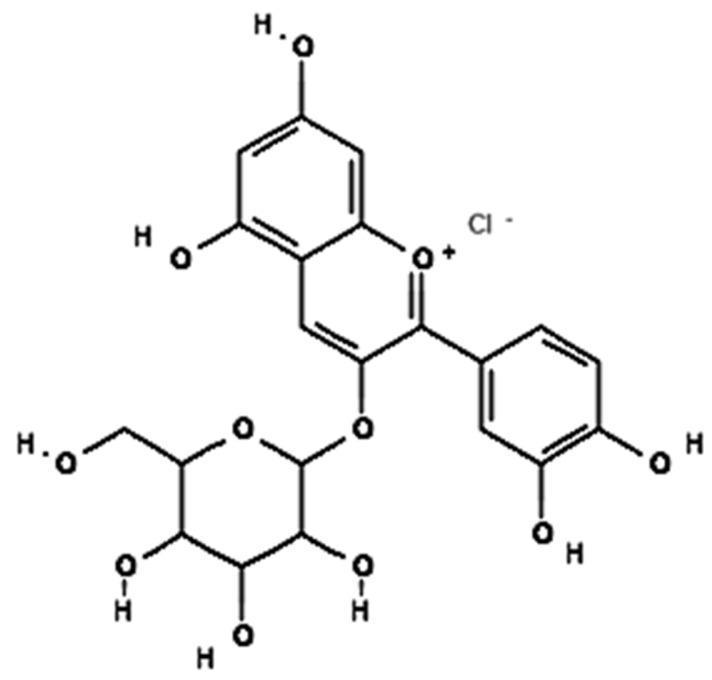
Chemical structures of potential of Cyanidin 3-glucoside as anti-obesity.

Cyanidin 3-glucoside (C3G) derivatives reduce obesity-induced inflammation by modulating adipocytokines secretion, and this action may be a useful strategy for preventing obesity-associated metabolic pathologies [205]. The bioavailability of cyanidin3-glucoside is only 0.02%, while the microbial degradation product of cyanidin 3-glucoside, 3,4-dihydroxybenzoic acid, has a bioavailability of 44% [206]. In an in vitro study, PC12 cells treated with H_2_O_2_ reduce pro-inflammatory markers associated with obesity, such as C-reactive protein (CRP), interleukin (IL)-6, and tumor necrosis factor (TNF)-α, leading to reduced oxidativestress-induced neurotoxicity in PC12 cells treated with H_2_O_2_ [202,207]. In vivo studies reported that the ovariectomized female Sprague–Dawley rats induced obesity by stimulating energy expenditure and modulation of lipid metabolism. The resultant activity showed a significant reduction in body weight gained by 32.83%, triglycerides by 24.4%, and LDL by 29.58%, compared to the control of the in vivo study [208]. In clinical trial studies in overweight and obese volunteers that observed changes in other markers of inflammation and lipid metabolism, plasma-oxidized LDL and serum malondialdehyde and hydroxynonenal concentrations decreased (NCT02613715) (Figure 8).

**Figure 9:**
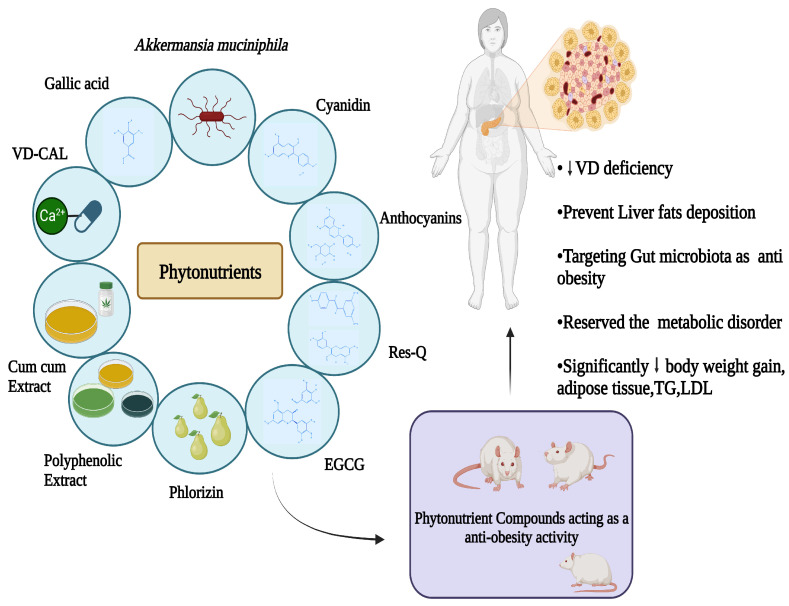
Phytonutrient mechanisms involved in obesity.

Sources of various phytonutrients, including their active components through various experimental models that are conducted in vivo studies as well as human-related, confirm their effect in the body system involving the fundamental mechanisms of action. The key findings that significantly show a response towards lowering body weight, fat accumulation or deposition, TG, LDL, and an alternative area to target obesity via targeting gut microbiota are shown in Figure 9 and Table 3.

## 5. Application of Phytonutrient-Based Anti-Obesity Food Supplement Products and Market Trends

**Figure 10:**
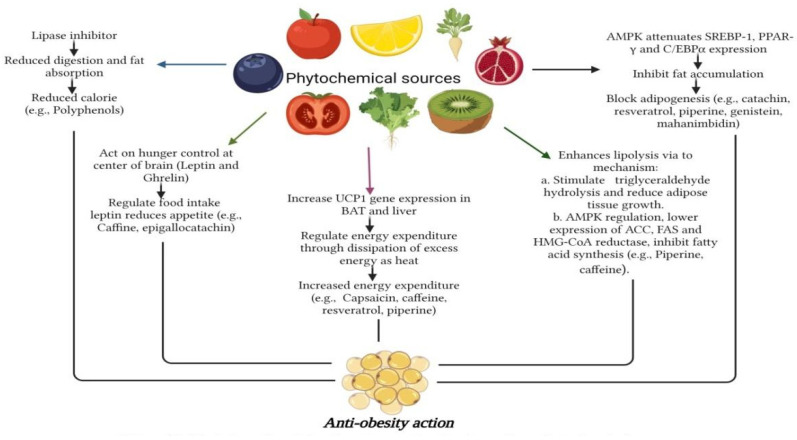
Depiction of anti-obesity action mechanism via phytochemicals.

In recent years, the increasing incidences of obesity have caught attention all over the world, and inadequate fat intake has been cited as one of the primary causes of obesity and its associated disorders. However, increasing investigation suggests that eating edible vegetable oils may have non-negligible physiological effects, such as reducing inflammation, decreasing blood lipids, and preventing the formation of adipocytes and hunger. Bioactive phytochemicals found in lipids and oils significantly affect obesity, such as phytosterols, phenolic compounds, and tocopherol [212]. In a study, obesity and metabolic syndrome have been linked to dietary intake of polyunsaturated fatty acids (PUFAs). Dietary n-3 PUFAs reduce leptin expression in adipose tissue and may prevent leptin resistance. This study further showed that n-3 PUFAs decrease leptin production, which is controlledby epigenetics, such as reduced methyl-binding protein 2 to the leptin promoter or via histone alteration within the promoter area [213]. Also, the impact of gastrointestinal hormones on fine-tuning hunger has been well-researched and is non-negligible. For example, PYY3-36 (PYY3-36),ghrelin,glucagon-like peptide-1 (GLP-1), pancreatic polypeptide (PP),oxyntomodulin (OXM),amylin, and cholecystokinin have all been demonstrated to impact food intake. Ghrelin enhances appetite and consumption of foods, but all other documented gut hormones have anorectic implications: they promote “satiation” (causing meal cessation) and/or “satiety” [214]. The Phytochemical Index (PI) and carbohydrate consumption had a beneficial correlation. Plant foods have a more beneficial form of carbohydrate than sweetened drinks, white bread, and other meals manufactured from refined flour. Furthermore, phytochemical-rich diets contain components that, like dietary fibers, can influence glucose metabolism. Dietary fibers can lower the glycemic index of meals, reducing glucose absorption and promoting lower insulin production, affecting more favorable physiological responses [215]. To examine the association between DPI (Dietary Phytochemical Index) and pre-diabetes morbidity, three hundred participants were surveyed and divided into 150 pre-diabetics (cases) alongside 150 healthy (controls) groups, and the DPI was determined based on data collected from a 168-item validated diet frequency questionnaire. Controlled cross-over research found that eating vegetables, whole grains, and fruits had a positive effect on FBG (fasting blood glucose) as well as insulin resistance in obese adults with increased FBG [216]. Figure 10 depicts the anti-obesity mechanisms offered by various phytochemicals.

Numerous epidemiologic as well as clinical studies have been conducted in recent years because of an increasing interest in preventing obesity with plant-based foods. These studies are crucial for expanding knowledge of the relationship between nutrition and obesity beyond its specific nutrients. According to a 2018 study by Mollica et al. [217], adding Turkish hazelnuts into a high-fat diet was linked to less weight gain, lower food intake, dose-dependent increases in triglycerides, TC, and HDL-C, a reduction in LDL-Cas well as atherogenic index, and a dose-associated reduction in plasma glucose concentration. The mice’s livers were not as affected by a high-fat diet biochemically and morphologically due to hazelnuts. Pomegranate-derived vinegar (PV) was studied by Samad, Azlan, and Ismail [218] and was shown to decrease lipogenesis and increase fatty acid beta-oxidation, making it an effective option for treating obesity. Additionally, consuming PV may increase the expression of the genes for the carnitine palmitoyl transferase 1 alpha (CPT-1a) and peroxisome proliferator-activated receptor alpha (PPARa), as well as phosphorylate AMPK more effectively than acetic acid, suggesting that PV is more effective than acetic acid in reducing obesity. According to Yun et al. [161], the CBVs (citrus-blended vinegars), using different blended ratios according to mandarin vinegar (MV), showed a dose-dependent reduction in intracellular triglyceride content. At 1/100 dilution, CBV2 had the lowest triglyceride content at 136.12 mg/dL, which was 65% lower than MV’s (209.82 mg/dL). Overall, in3T3-L1 cells, CBV was found to have anti-obesity effects by lowering intracellular triglyceride levels, lipid accumulation, and the mRNA levels of genes associated with adipogenesis and lipogenesis. Compared to mice receiving alcohol HFD, the intake of tomato wine enriched with lycopene showed a substantial protective effect, decreasing the buildup of visceral fat depots, such as the perirenal adipose and epididymal depots [219]. Through uncoupled oxidative phosphorylation, brown adipose tissue contributed to thermogenesis by encouraging lipid oxidation and thermogenesis instead of lipid storage. With the addition of tomato wine and lycopene, rats on the HFD gained noticeably browner adipose tissue weight [219]. The recently discovered tetrameric stilbenes (r- and r2-viniferin) were shown to be able to prevent adipocyte differentiation and decrease the total amount of lipids in 3T3-L1 cells by down-regulating the expression of the PPAR, C/EBP, and FABP4 genes. According to these findings, p21-(CDK inhibitor) and Rb-dependent regulation of transcription in 3T3-L1 cells showed thatr2-viniferin repressed the adipogenic process and prevented the cell cycle at the G1-S phase. Pterostilbene, a methylated resveratrol derivative, was reported to suppress the adipocyte development in both 3T3-L1 preadipocytes as well as 3T3-F442A cells when administered for a longterm at low doses (5–10 M) [220].

Along with plant secondary metabolites, dietary fibers also have been paid attention to for their positive correlation with obesity. In this regard, Sung et al. [221] developed a cereal *Allium fistulosum* extract bar (AFB) to determine the anti-obesity effect of HFD induced in obese mice. AFB therapy in HFD mice decreased body weight increase and glucose and raised blood triacylglycerol, lipid buildup in the liver, insulin levels, adipose tissue, and weight of adipose tissue. Adiponectin and blood HDL-cholesterol levels were also greater in the AFB groups than in the HFD group. In visceral adipose tissue, AFB therapy elevated the mRNA expression of receptor genes PPAR-c, UCP2, and b3-AR. Increased expression of these genes may act as a mediator in the reduction in total fat mass and adipocyte size and also reduce blood lipid levels caused by AFB therapy. Adipose tissue has high levels of PPAR-c expression, which is crucial for adipocyte development and fat storage in the liver. In order to increase fatty acid oxidation in WAT and reduce body weight, uncoupling protein-2, i.e., UCP2, is crucial. The stimulation of these receptors by specific agonists has significant anti-diabetes and anti-obesity benefits by inducing thermogenesis in response to prolonged lipolytic stimulation in rats, which nearly exclusively express b3-Adrenoreceptors (b3-AR) [221]. Recently, the novel biscuits utilized in the study were studied as an effective integrated approach to reducing obesity levels and associated impacts with date fiber (DF) as their primary target [222]. The rats in a diet that consumed biscuits supplemented with 10% DF had the lowest TGslevels, with no difference between them and the rats who ate biscuits with 15% DF, according to the results. The diet2 groups supplemented with 10% DF biscuits demonstrated the lowest levels of VLDL, with no appreciable variations from the orlistat supplement groups (G3). Additionally, other animal models fed biscuits enriched with differing DF levels (diet1 5% DFand diet210% DF) saw a drop in assessed LDL levels [222]. Gorjanović et al. [223] used mice in their report, which were subjected to normal food supplemented along with APF (apple pomace flour) and showed a remarkable reduction in body weight growth (about 39%). Apple polyphenol administration had a curative effect on body weight gain (BWG) and accumulation of fat, as well as enhanced tolerance for glucose in Wistar rats. According to one theory, AP controls the genes responsible for adipogenesis, lipolysis, and fatty acid oxidation. Similarly, in another study, lactic acid-producing bacteria, i.e., LAB, obtained from kefir, which is a popular probiotic drink, and wine grape seed flour (GSF), a prebiotic rich in polyphenols, are linked with metabolic disorders and obesity in (HFD)-induced obese mice. For 9 weeks, these mice were given HFDs containing 6% microcrystalline cellulose (CON), HFD enriched with GSF (5% or 10% GSF), HFD with LAB administered orally, or HFD with a combined dose of GSF and LAB administered orally (GSF+LAB). All GSF and LAB groups exhibited a decline in liver and adipose tissue weights, HF-induced weight gain, plasma lipid concentrations, glucose intolerance, and insulin resistance. Adipose tissue microarray data revealed that the interaction of GSF and LAB impacted genes related to immunological and metabolic disorders, including inflammasome complex assembly [224]. In obese mice induced by an HFD, grape seed flour boosted both energy expenditure (EE) as well as thermogenesis in BAT [225]. UCP1 along with additional metabolism-related genes were likewise impacted by GSF in WAT. The results obtained concluded that GSF reduced diet-induced obesity in mice (C57BL/6J) by improving energy metabolism. The increase in EE by encouraging BAT heat generation and WAT browning is the key factor contributing to the body weight reduction in the GSF-treated mice. Additionally, GSF may reduce several aspects of insulin resistance, glucose tolerance, and plasma biochemical indices. After high doses of GSF therapy, the expressions of various genes are linked to liver diseases, lipid metabolism, and changes in energy metabolism [225].

Recently, the kiwifruit jelly with chenpi (FKJ) designed with the addition of chenpi (30.26%), kiwifruit juice (35%), and pectin (2.88%) with the optimum mix via 3D printing revealed that dietary intake, adipose tissue, and liver weight decreased significantly after the intake of FKJ in a dose-dependent effect. When 3T3-L1 adipocytes are differentiated, it significantly decreases adipogenesis. Research has also shown that chenpi extract might successfully reduce obesity and hepatic steatosis brought on by an HFD [170]. Lim et al. [226] used germinated waxy black rice (GWBR) to assess the anti-obesity effect in 3T3-L1 adipocytes. The results indicated that the hot water extract from the germinated waxy black rice (GWBR) section had the greatest protective impact against lipid accumulation in 3T3-L1 adipocytes, increasing by 41% compared with control cells. GWBR extract lowered adipocyte proliferation and reduced lipid accumulation (more red staining). When 3T3-L1 adipocyte cells were exposed to 1 mg/mL concentration of GWBR extracted in hot water, it showed a substantial increase in expression of CPT-1 mRNA, which is linked to oxidation and UCP2 mRNA expression linked to thermogenesis. In another study, the positive benefits of tea and GTCs on obesity were determined [157]. The catechin EGCG was demonstrated as one of the most potent bioactive molecules among green tea catechins (GTCs). GTCs may slow digestive enzymes and limit absorption, thereby reducing fat. White adipose tissue accumulation and restrained body weight growth promote glucose absorption and increase glucose transporter-4 expression on muscle cell plasma membranes. As a result, green tea’s anti-obesity effects in HFD-induced obesity include overexpression of glucose transporter-4, reduction in absorption, and inhibition of digestive enzymes. In another report, a comparison of commercial anti-obesity medicine, orlistat (11.3%), was performed with SCOBY jackfruit drinks and suggested that jackfruit drinks significantly improved weight management control in HFD-fed obese mice and caused considerable body weight reduction (18.5–20.2%) [227]. Blood composition and signs of inflammation in medicated obese mice showed no negative consequences. Following the SCOBY jackfruit drinks diet treatments, gene expression involved in glucose transport, inflammatory cytokines, lipid biosynthesis, and chemokines in the adipose tissues was dramatically down-regulated. The sequencing of 16S rRNA from the mice’s feces demonstrated that SCOBY jackfruit drinks had changed the makeup of the gut microbiota, with the proliferation of helpful gut microorganisms being boosted in those animals relative to all control groups. Table 4 discusses a few studies where phytochemicals are used in the development of anti-obesity food products.

Consumers could overlook well-intended information without targeted, concentrated campaigns or efforts [235]. Because of this, lifestyle variables, including a healthy diet and exercise, continue to be crucial for preventing and treating obesity [236].

**Figure 11:**
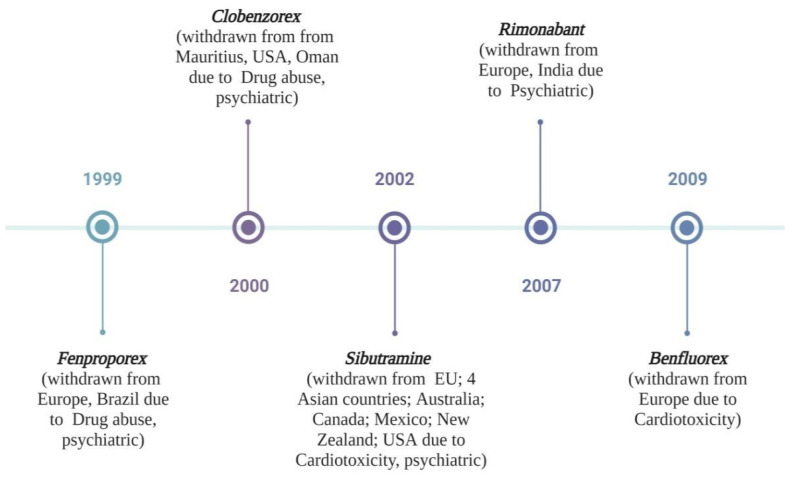
Health implications of anti-obesity drugs withdrawn from market.

Since there are limited pharmacological options on the market, there is an immediate need for new anti-obesity medications [237]. The majority of the anti-obesity medicines that were authorized and sold, yet, have since been pulled off the market because of dangerous side effects. Figure 11 depicts the recent timeline for the withdrawal of anti-obesity drugs from the market with their reported adverse effects. For example, fenfluramine and dexfenfluramine were pulled off the market in the 1990s due to their negative impact on heart valves. Due to an unfavorable risk-to-benefit ratio, the European Medicines Agency, or EMA, advised the removal of numerous anti-obesity medications from the market in 2000, including phentermine, diethylpropion, and mazindol. Currently, lorcaserin, phentermine, orlistat, naltrexone, and liraglutide are some of the anti-obesity active pharmaceutical components that have been authorized by the US FDA and are available on the market [238]. Only a small number of pharmaceuticals have been assigned for marketing purposes. Among those, some had to be pulled back due to serious side effects or widespread safety concerns with the approved therapies. This is despite significant research investments to find effective medications for treating obesity [239].

Regarding market division by application, one of the key contributors to the total sale of the nutritional product sector is the weight loss category. Over the projected period of 2015–2025, the growth of the weight loss or management category is predicted to rise at a compound yearly growth rate of about 7.4% [240]. Consumer views of the health-promoting properties of fruits, vegetables, and nuts—perceptions based on the phytonutrients found in these foods—have changed current market patterns. Phytonutrients are now added to, orextracted, orformulated in various food items and sold globally [241].

The impact of biotic and abiotic constraints, population income, technological and genetic advancement, the machinery revolution, faster access to information, the development of cities, the social and economic context, and climate changes that affect agricultural products and people’s movement from one region to another, all had a significant impact on agricultural market trends over the past ten years [242]. Indeed, phytonutrients are becoming more and more important in the market. The market for phytonutrients is predicted to develop at a compound annual growth rate (CAGR)of 7.2% between 2015 and 2020, reaching a potential value of USD 4.6 billion. Health problems, including cancer, type 2 diabetes, and cardiovascular illnesses, are the main driving factors for the worldwide phytonutrients market [243]. By dramatically boosting the intake of foods high in sugar, fat, salt, and saturated fats among children and adolescents, both food and beverage marketing in different channels and situations plays a crucial role [124]. The search for anti-obesity medications (AOMs) has become extremely difficult for both technological and societal reasons [244]. Many herbal products around the market make anti-obesity claims and are based on nutraceuticals. Nutraceutical anti-obesity supplements that are marketed include Sri Sri tattva, Jiva, Onelife amla, Lords, Dr. Bhargava, Medilexicon, and Onelife multiman [245]. The majority of anti-obesity medications have been demonstrated to enhance proxy measures of cardiovascular health, such as triglyceride concentration decreases, changes in blood pressure with weight loss, and elevations in HDL-cholesterol concentration [246]. Recent research found that the most often advertised natural weightloss compounds are chitosan, capsaicin, glucomannan, carnitine, and conjugated linoleic acid (CLA). Other widely used herbal compounds in Europe include the unroasted seeds of *Coffeaarabica* L. (Rubiaceae), *Garcinia cambogia* (Gaertn.) Desr., and *Camellia sinensis* (L.) Kuntze (Theaceae) [247].

Several problems were encountered when applying phytochemicals as an anti-obesity supplement/drug. The key reasons that they are frequently citedare poor stability, minor bioavailability, low water solubility, and rapid breakdown by enzymes in the digestive system, liver, kidneys, and other organs. When these phytochemicals are encapsulated, sufficient amounts may be supplied to the desired organs and cells, promoting fat loss and enhancing general metabolic status. These preliminary results, however early, may signal the beginning of a new approach to dealing with the increasing incidence of obesity that may also behighly compliant [136]. Another problem is that hand-made herbal medicines have no way of being controlled or registered, and many nations have poorly regulated markets for them. Many stakeholders have reported multiple instances of adulteration of herbal weight reduction medications. Research of 160 herbal food weight reduction supplements showed that more than 50% had six APIs (active pharmaceutical ingredients) together. In herbal medicines, unreported APIs for sibutramine, sildenafil, phenolphthalein, fluoxetine, and lorcaserin were found [248]. Anti-obesity herbal drugs, including those sold in stores and online, can vary greatly in composition. They have unpredictable high concentrations of active substances and unpredictably negative consequences. In addition to having variable quantities of active chemicals, these products can have unpredictable and potentially hazardous results. They could include adulterants, such as discontinued medications and drug mimics as well as thyroid extracts (e.g., fenfluramine). For all the aforementioned reasons, herbal anti-obesity treatments may directly be harmful or interact negatively with other drugs. Researchers, doctors, and other medical experts need to be conscious of the issue. They need to caution their patients of the possible hazards of using these drugs and their diverse character. Certain drugs and some substances in dietary supplements for weight loss may interact. When combined with other stimulants, coffee and bitter orange, for instance, this may have an additional impact. It has been demonstrated that bitter orange inhibitstheCYP3A4 function, raising blood levels of certain medications like cyclosporine and saquinavir (National Institute of Health, accessed on 7 April 2023). To boost product research and development, market participants are concentrating on consolidation and cooperation methods.

## 6. Future Perspectives and Conclusions

The rising global prevalence of obesity has led to a pressing need for effective preventive strategies. In this context, phytonutrients have emerged as promising candidates for anti-obesity prevention. Phytonutrients are bioactive compounds in plant-based foods that exhibit various biological activities, including antioxidant, anti-inflammatory, and metabolic modulation properties. These compounds have the potential to influence key mechanisms involved in the development and progression of obesity, making them attractive targets for preventive interventions.

One future perspective is further exploring the molecular mechanisms through which phytonutrients exert their anti-obesity effects. Understanding the specific pathways and targets that phytonutrients modulate can provide valuable insights into their potential as preventive agents. This knowledge can guide the development of targeted interventions that optimize the use of phytonutrients for anti-obesity purposes [249].

Another important aspect of future perspectives is utilizing food waste and by-products to maximize the potential of phytonutrient sources [250,251]. Many phytonutrient-rich parts of plants, such as peels, stems, and leaves, are often discarded as waste. However, these parts can be valuable sources of phytonutrients and bioactive compounds. Innovations in food processing technologies and developing novel food products can help extract and incorporate these phytonutrient-rich components into the regional diet, reducing waste and improving nutritional value.

In conclusion, the future perspective of phytonutrients as anti-obesity prevention is promising. Further research is needed to elucidate the underlying molecular mechanisms, identify specific phytonutrients with potent anti-obesity properties, develop innovative delivery systems, and explore synergistic effects with other bioactive compounds. Harnessing the potential of phytonutrients in preventive interventions can contribute to global efforts to combat obesity and promote public health. The development of innovative food supplement products and functional foods incorporating phytonutrients can offer convenient and effective ways to deliver these bioactive compounds to consumers.

## Figures and Tables

**Table 2:** Effects of various phytonutrients on obesity (in vitro, animal study, and human study).

Phytonutrients/ Source	Experimental Model/Condition	Experimental Design/Trial	Main Outcomes	References
Red ginseng (saponin fraction)	3T3-L1 and RAW264.7 cells	Cells (1 × 10^4^ or 1 × 10^5^ cells/well) were incubated in 96-well plate for 24 h, sample (SF) (100 μg/mL) pretreated for 2 h and incubated for 24 h or 48 h; medium was removed and MTT reagent was added for 60 min at 37 °C followed by Dimethyl sulfoxide (DMSO) was added; and absorbance was recorded at 550 nm	SF suppressed tumor necrosis factor-alpha (TNF-α) (78%), monocyte chemo attractant protein-1 (MCP-1) (40%), and interleukin-6 (IL-6) (22%). Increases nuclear factor erythroid-derived 2(Nrf2) and target protein, hemoxygenase-1 (HO-1).	[195]
11 protoberberine-type alkaloids (obtained from *Berberis* (*Berberidaccae*) and *Coptis* rhizomes)	3T3-L1 adipocytes	Alkaloids (5µM) treated on adipocytes for 4 days, followed by adipocyte staining with Oil Red O on Day 12	13-Methylberberine down-regulated the expression of adipocyte differentiation transcription factors, (PPARγ) and CCAAT (C/EBPα).	[180]
Zeaxanthin	3T3-L1 adipocytes	Cells were seeded in 96-well plates at 1 × 10^3^ densityandwere cultured for 12 h in serum-free DMEM, various concentrations of zeaxanthin (0–60 µM) were incubated for 72 h;cell toxic effects of zeaxanthin were evaluated	Zeaxanthin significantly decreases the lipid content from intracellular in dose-dependent manner (5–15 µM) in adipocytes without causing cytotoxicity.	[190]
Green tea	High-fat diet-fed rats	400 to 800 mg/kg dose was given for 6 weeks	Low serum leptin levels in rats; Inhibits fatty acid absorption; Suppresses expression of both IL-6 as well as TNF-α gene.	[156]
Adzuki beans (*Vigna angularis* L.) flavonoids and saponins	ABTE (Adzuki bean total extract), ABF (Adzuki bean flavonoid), and ABS (Adzuki bean saponin) orally administered in HFD mice	60 and 300 mg/kg per day ABTE, ABF, and ABS for 4 weeks	Enhance lipolysis; reduce final body weight and adipose tissue accumulation. Reduce total cholesterol, serum triglyceride levels, LDL-cholesterol, and liver lipid.	[199]
Flavonol kaempferol	Male mice C57BL/6J	HFD+kaempferol @ 0.01 or 0.05% for 5 months	Increased lipolysis. Prevents high fatty acid-impaired glucose uptake, glycogen synthesis, AMPK activity, and Glut4 expression in skeletal muscle cells. Improving peripheral insulin sensitivity and protecting against pancreatic *β*-cell dysfunction.	[153]
Muscadine grape or wine	Male C57BL/6J mice	Low-fat diet (LF, 10% kcal fat) High-fat diet (HF, 60% kcal fat) HF + 0.4% muscadine grape phytochemicals (HF+MGP) HF + 0.4% muscadine wine phytochemicals (HF+MWP) for 15 weeks	Decreased body weight by 12% compared to HF controls. Reduced plasma content of triglycerides, free fatty acids, and cholesterol in obese mice.	[154]
Sprouted Red Radish seed	Low-calorie diet with sprouted red radish seed (100 g per day) in adult female (25–40 years old) for 8 weeks	Anthropometric measurements and blood samples for analysis	Suppressed the elevations in body weight (~12%), serum cholesterol (~27.5% reduction), serum triglycerides (~33% reduction), and glucose (~7% reduction).	[200]
Tomato	35 Caucasian women aged between 18 and 25 years consume tomato (cv. rama) @ average of 90 g/per day before lunch for 4 weeks	Anthropometric and biochemical parameters were measured	Reduced body weight, blood glucose, % fat, cholesterol, fasting triglycerides, and uric acid.	[191]

**Table 3:** Phytonutrients in various experimental models with their major findings in obesity.

Phytonutrient Compounds	Mechanism System Target	Experimental Model	Major Findings	References
Phlorizin (PHZ)	In vitro study: Block the metabolic;LDL oxidation was prevented.	LDL-C isolated from human plasma	LDL-cholesterol oxidation was blocked	[158,159]
	In vivo study: Improved manufacturing of the hormone glucagon-like peptide-2 (GLP-2) and healing of gut epithelial barriers disruption brought on by HFD. Clinical trial:Not yet	High-fat diet (HFD) induced gut microbiota alterations	PHZ’s anti-obesity action may also be mediated through the gut microbiota–barrier pathway.	
Epigallocatechin gallate	In vitro study: Reduce adipogenesis to lower C. elegans fat content, as indicated by the reduced ATGL-1 gene expression level following EGCG therapy.	*C. elegans* strains and *Escherichia coli*OP50diet	Controls the body fat content	[163,165,209]
	In vivo study: Increasing thermogenesis to improve utilization of energy.	Male C57BL/6J mice induced obesity	The blood sugar and triglyceride concentrations were significantly decreased, lowering lipid formation in adipose tissues and affecting weight growth.	
	Clinical trial: Controlled the plasma cholesterol and triglycerides	15 women with central obesity were screened at our clinic. A total of 102 of them with a body mass index (BMI) ≥ 27 kg/m^2^ and a waist circumference (WC) ≥ 80 cm	Decrease body weight and BMI in obese women after a 12-week treatment, significantly decrease waist circumference.	
Anthocyanins	In vitro study: Anthocyanins reduce the complete downstream cascade of pro-inflammatory mediators, including C-reactive protein (CRP), interleukin (IL)-6, and tumor necrosis factor (TNF), and they also ameliorate gut dysbiosis, therefore, reestablishing a healthy gut microbiota.	IL-6 gene in lipopolysaccharide (LPS)-induced adipose stem cells	Treat obesity-related inflammation and chronic diseases.	[202,203,204]
	In vivo study: Regulating neuropeptide Y, and the -aminobutyric acid receptor in the hypothalamus decreases appetite	Male Sprague–Dawley rats induced obesity	In comparison to the control, there was a substantial decline in body weight growth (15.76%) and regular caloric consumption (19.10%)	
	Clinical trial: Lowered the incidence of metabolic disorders and decreased lipids, body composition, and inflammation in obese people	Healthy male volunteers	Reduce body mass index, low-density lipoprotein cholesterol	
Resveratrol and Quercetin	In vitro study: Lower the gene expression of the essential adipogenic factors peroxisome proliferator-activated receptor (PPAR) and CCAAT/enhancer binding protein (C/EBP) to suppress adipogenesis.	Human SGBS adipocytes	Reduced concentrations of the adipokines ANGPTL4, adipsin, and PAI-1 as well as the glycolysis-related enzymes ENO2, PFKP, and PFKFB4; all arelinked to obesity and malfunction of adipose tissue.	[173,174]
	In vivo study: Alteration of the intestinal flora	Male Wistar rats induced obesity	Reductions in adipocyte size, visceral adipose tissue weight, and body weight increase that are substantial	
	Clinical Trial: The inhibition of genes related to angiogenesis, Wnt signaling, intercellular connection, G protein-coupled receptors, and Notch signaling mechanisms involved in cell cycle regulation that have been up-regulated	11 obese otherwise healthy men	Adipogenesis increased and adipocyte size was decreased.	
Gallic acid	In vitro study: The size of adipocytes effected due adipose tissue inflammation and metabolic dysfunction, which are linked to adipocyte hypertrophy	Murine 3T3-L1 preadipocytes and RAW 264 macrophages	A typical fat cell content, the WAT group’s 18 size was noticeably smaller as compared to the GA group; with a control group	[169,210,211]
	In vivo study: Lipolysis is induced and FAS is suppressed to prevent lipogenesis as a means of controlling the process of lipid metabolism.	Male C57BL/6 mice induced obesity	Weight loss and a decline in the accumulation of fat	
	Clinical trial: Suppressing adipogenesis and proliferation and reducing pancreatic lipase activity	Obese human subjects receiving capsules containing 200 mg of gallic acid and 50 mg of a Chinese herbal decoction, three times a day for 24 weeks	Serum levels in humans, which is what prevented it from causing weight reduction or a decrease in food intake.	
Cyanidin	In vitro study: Lower the levels of pro-inflammatory substances correlated with fat, including tumor necrosis factor (TNF), interleukin-6, and C-reactive protein (CRP)	PC12 cells treated with H_2_O_2_	In PC12 cells exposed to H_2_O_2_, oxidative stress-associated toxic effects were reduced.	[202,208]
	In vivo study: Regulates the breakdown of lipids and enhancement of energy consumption	Ovariectomized Female Sprague–Dawley rats induced obesity	Significantly reducing the rate of obesity increase by 32.83%, triglycerides by 24.4%, and LDL by 29.58%, contrasted to control	
	Clinical trial: Alterations to lipid metabolism in conjunction with inflammatory indicators	Overweight and obesity (250 mL of blackberry juice)	Reduced levels of malondialdehyde, hydroxynonenal, and serum-oxidized LDL in the plasma and serum	

**Table 4:** Application of phytonutrient-based anti-obesity food supplement products.

Phytonutrients/ Source	Food Application and Purpose	Functional Effect Claim	Major Findings	References
Blueberry anthocyanin (BA)	Obese mice were supplemented with BA in daily food at doses of 50, 100, and 200 mg/kg	Anti-obesity effect	− Decrease serum glucose, attenuate epididymal adipocytes, and improve lipid profiles. − Expression of TNFa, IL-6, PPARg, and FAS genes were down-regulated.	[228]
Allicin (vinyldithinins, sulfides, and ajoene)	Dates fruit and garlic-based beverage	Anti-obesity	− Ajeone has been noted to mediate apoptosis and reduce lipid accretion in adipocytes 3T3-L1.	[229]
Total polyphenols and monosaccharides	Tremella and blueberryfermented concentrate	Anti-obesity	− Modulated the diversity of intestinal microbiota. − Improved blood lipid profiles. − Reduced overall body weight.	[230]
Nipa vinegar	Nipa vinegar added in mice HFD-fed at 0.08 and 2 mL/kg body weight for 33 weeks	Anti-inflammatory and anti-obesity effect	− Reduced lipid deposition. − Improved the serum lipid profile. − Increased adipokine expression. − Suppressed inflammation.	[231]
Gingerol from ginger	Supplementation of Gingerol (25–75 mg/kg) suspended in 0.5% carboxy methylcellulose with high-fat diet of mice	Anti-obesity	− Reduced glucose level, leptin, amylase, insulin, lipase plasma, tissue lipids, and body weight	[232]
Dietary fiber from bamboo shoot	Bamboo shoots lyophilized powder	Anti-obesity	− Reduced 30.56% body weight. − Improved insulin resistance and inflammation in obese mice. − Increased both short-chain fatty acids levels and SCFA-producing microbes.	[233]
Broccoli microgreens juice	20 g/kg/body weight broccoli microgreen juice was given in high-fat and high-sugar mice diet for 2 to 10 weeks	Reduce obesity in mice	− Reduced white adipose tissues (WATs) mass, adipocyte size, and the body weight. − Increased water intake in HFD-fed mice. − Improved glucose tolerance and HOMA-IR value. − Reduced insulin level and alleviated insulin resistance.	[234]

## Data Availability

Not applicable.
